# AQP7 deficiency drives adipose tissue remodeling and disrupts homeostasis

**DOI:** 10.1038/s44324-025-00085-y

**Published:** 2025-11-07

**Authors:** Ines PD Costa, Guglielmo Schiano, Juan Manuel Sacnun, Rebecca Herzog, Alastair Kerr, Ingrid Dahlman, Christine Delporte, Klaus Kratochwill, Olivier Devuyst

**Affiliations:** 1 IREC, UCLouvain, Brussels, Belgium; 2 Institute of Physiology, University of Zurich, Zurich, Switzerland; 3 Division of Pediatric Nephrology and Gastroenterology, Department of Pediatrics and Adolescent Medicine, Comprehensive Center for Pediatrics, Medical University of Vienna, Vienna, Austria; 4 Department of Medicine (H7), Department of Clinical Science and Education, Karolinska Institutet, Södoroersjukhuset, Stockholm, Sweden; 5 Laboratory of Pathophysiological and Nutritional Biochemistry, Faculty of Medicine, Université Libre de Bruxelles, Brussels, Belgium; 6 Cliniques Universitaires Saint-Luc, Brussels, Belgium

**Keywords:** Endocrine system and metabolic diseases, Metabolic disorders, Metabolism, Metabolic syndrome, Obesity

## Abstract

The aquaporin-7 (AQP7) channel mediates glycerol release from adipocytes. Genetic variants decreasing AQP7 expression are associated with adiposity and metabolic complications in humans. Using human data, mouse models, and cellular systems, we investigated how AQP7 influences adipose tissue maturation and homeostasis. Negative correlations between methylation on the *AQP7* locus, expression of *AQP7* in the adipose tissue and BMI were observed in humans. Mice lacking *Aqp7* had increased body weight and visceral fat accumulation, due to adipocyte hypertrophy and chronic inflammation, impairing transport across the peritoneal membrane. These changes were further intensified by a high-glucose diet. Mechanistically, AQP7 deficiency disrupted the expression of genes related to adipogenesis and adipocyte function, resulting in a shift toward fibrosis and inflammation, while secreted factors from AQP7-null adipocytes promoted fibroblast activation. These findings establish AQP7 as a key regulator of adipose tissue homeostasis, metabolic dysregulation, and inflammation/fibrosis, exacerbated by glucose-induced obesity.

## Introduction

Aquaporin-7 (AQP7) is a prototypical aquaglyceroporin expressed in mature adipocytes, where it facilitates the selective transport of water and glycerol across cell membranes, supporting glycerol efflux and maintaining energy homeostasis^1,2^. Genetic deletion of AQP7 in mice resulted in glycerol accumulation, adipocyte hypertrophy, adult-onset obesity, insulin resistance and altered glycerol metabolism^3–5^. In humans, common variants in the promoter region of *AQP7*, linked to a reduced expression of *AQP7* in the adipose tissue, have been associated with obesity and metabolic complications^6–8^. Despite these associations, the mechanisms through which AQP7 influences adipocyte biology and maturation, as well as its role in body weight gain and metabolic complications remain poorly defined.

Recent studies show that AQP7 is predominantly expressed in the visceral adipose tissue (VAT) of both mice and humans, where its expression can be regulated by metabolic cues such as fasting^9,10^. The VAT, which surrounds internal organs within the abdominal cavity, is a highly metabolically active depot essential in lipid storage and endocrine signaling^11–13^. However, VAT is also particularly susceptible to dysfunction in the context of metabolic stress. Alterations in VAT, particularly hypertrophy of adipocytes, disrupts normal adipose tissue function and contributes to the development of metabolic disease^14^. Hypertrophic adipocytes secrete elevated levels of cytokines and chemokines —such as MCP-1, IL-6, and IL-1β—which drive immune cell infiltration and promote chronic, low-grade inflammation^14,15^. This inflammatory response contributes to impaired insulin sensitivity and to adverse tissue remodeling. Dietary factors, particularly high intake of added sugars through sugar-sweetened beverages, promote VAT expansion, adipocyte hypertrophy, and ectopic fat accumulation^16–18^. Chronic high glucose exposure, e.g., excessive sugar intake, may influence the expression and function of AQP7, further modulating adipocyte phenotype and tissue remodeling^19^. Understanding the role of AQP7 in the context of VAT expansion and inflammation may provide important clues for uncovering mechanisms of adipose tissue remodeling and metabolic dysfunction.

The relationship between AQP7 and adipose tissue is especially relevant in the setting of peritoneal dialysis (PD), where patients are exposed daily to glucose-rich dialysis solutions to facilitate solute and water transport across the peritoneal membrane^20^. During PD exchanges, glucose from the dialysate is quickly absorbed into the systemic circulation^21^. Over time, this results in systemic absorption of large amounts of glucose—estimated at 40 to 100 kg per year^22^—equivalent to a chronic high-sugar exposure. Such hyperglycemic stress may promote VAT expansion, drive adipocyte hypertrophy, and disrupt normal adipose tissue architecture. These changes contribute not only to local peritoneal membrane deterioration but also to systemic metabolic disturbances, including insulin resistance and chronic inflammation. Hence, the impact of prolonged glucose exposure in PD extends beyond fluid transport, as peritoneal membrane alterations are associated with mortality rates that are up to 20 times higher than the general population, with cardiovascular disease accounting for ∼60% of PD-related deaths^20,23^.

This study explores the role of AQP7 in the VAT remodeling and dysfunction, using mouse and cellular studies complemented by human data. Correlations between AQP7 methylation, its expression in the adipose tissue, and BMI were observed in humans. In mice, *Aqp7* deficiency led to visceral fat accumulation, inflammation, and impaired peritoneal transport due to adipocyte hypertrophy. Mechanistically, AQP7 deficiency impaired adipogenesis and adipocyte function, leading to changes in secretory profiles and tissue remodeling. These findings establish AQP7 as a key regulator of adipose homeostasis and a potential therapeutic target for reducing fat accumulation and metabolic imbalances associated with high glucose exposure.

## Results

### Epigenetic regulation of *AQP7* links adipose tissue expression and obesity in humans

**Fig. 1:**
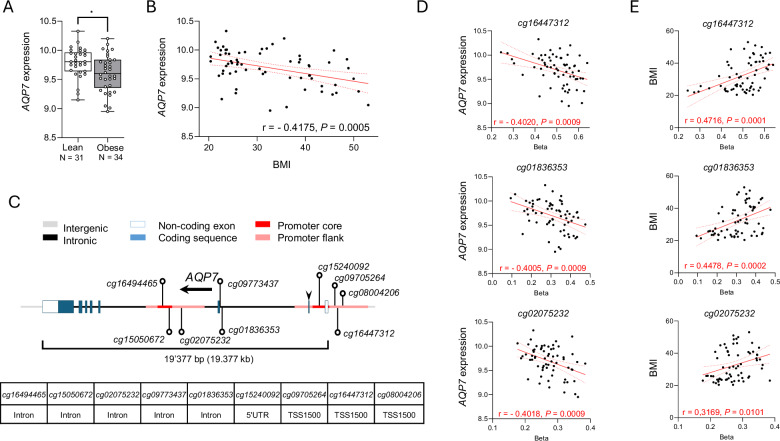
Expression of *AQP7* and methylation of the *AQP7* locus in adipose tissue in a cohort of women spanning a range of BMI. **A** Obese individuals (BMI >30.0 kg/m^2^; *n* = 34) exhibit significantly lower *AQP7* expression in the visceral adipose tissue than lean individuals (*n* = 31). **B** Negative correlation between the level of *AQP7* expression and the BMI in the cohort of 65 women. **C** The *AQP7* gene is composed of eight exons that span more than 19 kb. The nine identified methylation sites in the *AQP7* locus are indicated, four in the promotor region and five in the intronic regions. **D-E** DNA methylation analysis reveals that seven sites correlate negatively with *AQP7* expression (**D**), with three of these sites positively correlating with BMI (**E).** Significant differences between groups were assessed by *t*-test : *p < 0.05, **p < 0.01, ***p < 0.001.

Common genetic variants in *AQP7*, associated with a reduced expression of the channel in adipocytes, have been associated with obesity^6^. To substantiate the influence of AQP7 on adipose tissue in humans, we first investigated the expression of *AQP7* in a cohort of 65 women with a median BMI of 30.6 kg/m^2^ (IQR:23.9–40.8). Individuals in the obese group (*N* = 34, BMI >30.0 kg/m^2^) had a significantly lower *AQP7* expression in the VAT when compared to lean individuals (*N* = 21, BMI <25 kg/m^2^) (Fig. 1A). A significant, inverse correlation (*r* = −0.4175, *P* < 0.001) between the level of *AQP7* expression in the VAT and BMI was identified in the cohort (Fig. 1B).

In humans, the *AQP7* gene is located on chromosome 9 and is composed by eight exons spanning 19 kb (Fig. 1C). We analysed the degree of DNA methylation of nine methylation sites within *AQP7*, four in the promoter region and five in intronic regions. Seven of these methylation sites showed a significant negative correlation with *AQP7* expression levels in the VAT (Fig. 1D, Supplementary Table 2, and Supplementary Fig. 2); three of these sites exhibited a positive correlation with BMI (Fig. 1E).

Together, these data demonstrate a close, inverse relationship between AQP7 expression, influenced by methylation of the *AQP7* locus, and BMI in humans. This suggests that a decreased expression of AQP7 may contribute to fat accumulation and body mass index.

### Progressive metabolic and adipose tissue dysfunction in *Aqp7*-deficient mice at baseline and in response to glucose exposure

**Fig. 2:**
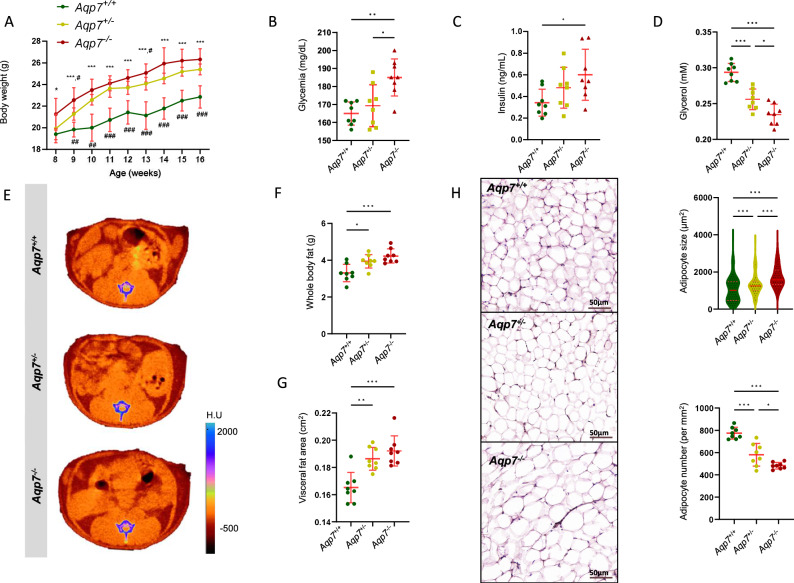
Impact of AQP7 deficiency on body weight and composition, metabolic parameters, and adipose tissue in mouse. **A** *Aqp7*-deficient mice exhibit a significant progressive increase in body weight from 8 to 16 weeks, compared to their wild-type littermates. **B**–**D** The deletion of *Aqp7* is reflected by **B** elevated fasting glycemia and **C** increased insulin levels, both showing a dose-dependent rise in *Aqp7*^−/^^−^ mice. In contrast, **D** plasma glycerol levels, progressively decline in *Aqp7*^+/−^ and *Aqp7*^−/−^ mice, reflecting the level of AQP7 deficiency. **E**–**G** Compared to their wild-type counterparts, *Aqp7*-deficient mice demonstrate a marked increase in **F** whole-body fat and **E**–**G** visceral fat accumulation, both of which increase progressively with AQP7 deletion, with more pronounced effects in *Aqp7*^*−/−*^ mice. **H** The deficiency in *Aqp7* is further associated with a progressive reduction in adipocyte number and a corresponding enlargement in adipocyte size with increasing AQP7 deficiency. Significant differences between groups were assessed using ANOVA followed by Tukey’s multiple comparisons test: *p < 0.05, **p < 0.01, ***p < 0.001.

To substantiate the role of AQP7 in VAT, BMI and related functional parameters, we investigated the effect of a genetic modulation of AQP7 in *Aqp7* mice over time (8 to 16 weeks of age) under standard diet (Table 1 and Fig. 2). At 8 weeks of age, AQP7 knock-out (*Aqp7*^*−/−*^) mice displayed a significantly higher body weight than their wild-type (*Aqp7*^+/+^) littermates, with intermediate values in the heterozygous *Aqp7*^+/−^ littermates. The differences between genotypes were amplified at 16 weeks (Table 1), with both *Aqp7*^*+/−*^ *and Aqp7*^*−/−*^ mice showing significantly increased body weight compared to *Aqp7*^+/+^ littermates (Fig. 2A). These changes were paralleled by a significant increase in glycemia (Fig. 2B) and serum insulin (Fig. 2C) levels in *Aqp7*^−/−^ mice. Conversely, serum glycerol levels were significantly lower in both *Aqp7*^+/−^ and *Aqp7*^−/−^ mice (Fig. 2D), whereas the levels of free fatty acid were similar between all groups. The changes in body composition were confirmed using a micro-CT scan (Fig. 2E): when compared to *Aqp7*^*+/+*^ mice, both *Aqp7*^+/−^ and *Aqp7*^−/−^ mice exhibited increased fat accumulation (Fig. 2F), particularly visceral fat (Fig. 2G). The level of AQP7 deletion was reflected by a progressive reduction in adipocyte number and a parallel enlargement of their size (Fig. 2H). Of note, *Aqp7*^*−/−*^ mice showed higher ectopic fat deposition with increased fat infiltration in both muscle and liver (Table 1).

**Table 1:** Impact of AQP7 deficiency on body weight and metabolic and adipose tissue parameters in mouse

	*Aqp7*^+/+^	*Aqp7*^+/−^	*Aqp7*^−^^/−^
Body weight (g)	22.9 ± 1.0	25.4 ± 0.5^***^	26.3 ± 1.0^***^
Metabolic parameters (Plasma)			
Glucose (mg/dL)	165.0 ± 6.5	169.4 ± 11.6	185.0 ± 10.3^**$^
Insulin (ng/mL)	0.34 ± 0.12	0.48 ± 0.19	0.60 ± 0.24^*^
Glycerol (nM)	0.29 ± 0.01	0.26 ± 0.01^***^	0.23 ± 0.01^***$^
Free fatty acid (nM)	0.45 ± 0.12	0.36 ± 0.09	0.34 ± 0.12
BUN (mg/dl)	28.4 ± 4.7	28.6 ± 2.8	31.4 ± 4.4
Adipose tissue parameters			
Whole-body fat (g)	3.30 ± 0.48	3.95 ± 0.37^*^	4.24 ± 0.39^***^
Visceral fat area (cm^2^)	0.17 ± 0.01	0.19 ± 0.01^**^	0.19 ± 0.01^***^
Dorsal muscle density	0.92 ± 0.03	0.90 ± 0.03	0.88 ± 0.03^*^
Liver density	0.93 ± 0.03	0.91 ± 0.02	0.90 ± 0.02^*^
Adipocyte size (μm^2^)	1073 ± 668	1335 ± 577^***^	1728 ± 573^***$$$^
Adipocyte number	774 ± 53	580 ± 102^***^	482 ± 29^***$^

Results are mean ± SD values; *n* = 8 mice per group (age 16 weeks).

The differences between groups were assessed by ANOVA followed by Tukey’s multiple comparisons test. The significance level is indicated as: ^*^p < 0.05; ^**^p < 0.01; ^***^p < 0.001 versus *Aqp7*^+/+^; ^$^*p* < 0.05; ^$$^*p* < 0.01; ^$$$^*p* < 0.001 versus *Aqp7*^+/−^.

*BUN* blood urea nitrogen.

**Fig. 3:**
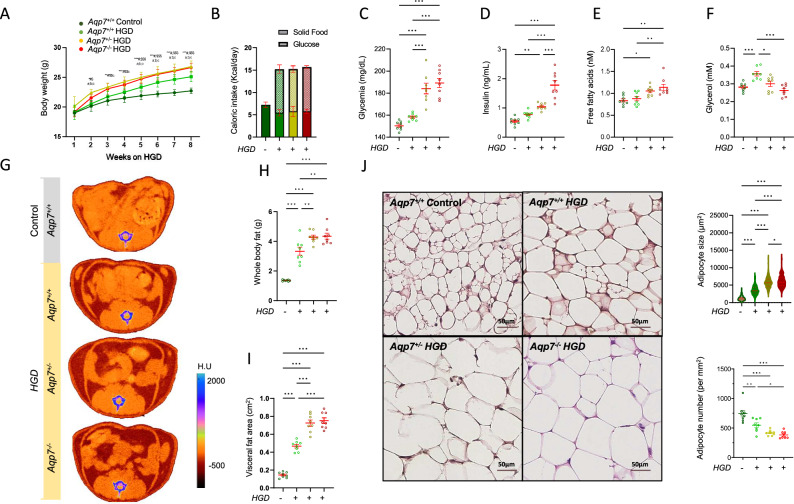
Impact of AQP7 deficiency and high glucose diet on body weight and composition, metabolic parameters, and adipose tissue in mouse. **A** Mice receiving glucose-supplemented water for 8 weeks exhibit a significant increase in body weight, which was particularly pronounced in *Aqp7*-deficient mice. **B** This weight gain in *Aqp7*-deficient mice is not due to increased caloric intake, as no significant differences are observed in caloric consumption between genotypes. The deletion of *Aqp7* is associated with **C** elevated fasting glycemia, **D** increased insulin levels, and **E** higher free fatty acid levels in both *Aqp7*^*+/−*^ and *Aqp7*^*−/−*^ mice, all showing a dose-dependent rise. In contrast, **F** plasma glycerol levels progressively decline in *Aqp7*^+/−^ and *Aqp7*^−/−^ mice, reflecting the extent of AQP7 deficiency. **G**–**I** Micro-CT imaging reveal significant increases in **H** whole-body fat and **I** visceral fat area in *Aqp7*-deficient mice following glucose intake, with the most pronounced effects in *Aqp7*^*–/−*^ mice. **J** AQP7 deficiency is further linked to a progressive reduction in adipocyte number and a corresponding enlargement in adipocyte size with increasing AQP7 deficiency. Significant differences between groups were assessed using ANOVA followed by Tukey’s multiple comparisons test: *p < 0.05, **p < 0.01, ***p < 0.001.

We next investigated whether genetic modulation of AQP7 influences the effect of high glucose exposure (8 weeks) on body composition and metabolic parameters (Table 2 and Fig. 3). All mice receiving the high-glucose diet showed an increase in body weight (Fig. 3A). Notably, this increase in body weight was more pronounced in *Aqp7*^−/−^ mice compared to wild-type *Aqp7*^*+/+*^, unrelated to increased caloric intake (Fig. 3B). Both haplodeficient and knock-out *Aqp7* mice showed increased fasting glycemia, insulin and free fatty acid serum levels (Fig. 3C–E), while their levels of serum glycerol were decreased (Fig. 3F). Micro-CT imaging revealed that the combination of AQP7 deficiency and glucose consumption resulted in a notable increase in whole body and visceral fat (Fig. 3G–I), combined with increased fat infiltration in both muscle and liver (Table 2). The *Aqp7* genotype had also a substantial impact on adipocyte number and adipocyte size (Fig. 3J), confirming the dose-dependent relationship between AQP7 levels and adipocyte enlargement.

**Table 2:** Impact of AQP7 deficiency on body weight and metabolic parameters after high glucose diet in mouse

		High-glucose diet (8 weeks)		
	*Aqp7*^*+/+*^	*Aqp7*^*+/+*^	*Aqp7*^*+/−*^	*Aqp7*^*−/−*^
Body weigh after diet (g)	22.7 ± 0.5	25.1 ± 0.9^***^	26.8 ± 1.0^***$$$^	26.7 ± 0.7^***$$^
Body weight gain (%)	19.6 ± 3.9	30.63 ± 4.2^***^	33.6 ± 8.2^***^	39.13 ± 3.7^***$$^
Kcal intake (Kcal/day)	7.23 ± 0.79	15.20 ± 1.96	15.32 ± 2.55	15.68 ± 0.83
Kcal food intake (Kcal/day)	7.23 ± 0.79	5.45 ± 1.44	5.87 ± 2.22	5.77 ± 0.89
Kcal glucose intake (Kcal/day)	0.00 ± 0.00	9.75 ± 0.95	9.45 ± 0.73	9.90 ± 0.42
Metabolic parameters (plasma)				
Glucose (mg/dL)	150.4 ± 4.0	158.1 ± 3.0	184.1 ± 13.6^***$$$^	189.3 ± 11.9^***$$$^
Insulin (ng/mL)	0.55 ± 0.13	0.78 ± 0.12	1.05 ± 0.11^**^	1.78 ± 0.46^***$$$###^
Glycerol (nM)	0.28 ± 0.02	0.36 ± 0.03^***^	0.30 ± 0.04^$^	0.26 ± 0.03^$$$^
Free fatty acid (nM)	0.83 ± 0.11	0.89 ± 0.15	1.05 ± 0.11^*^	1.14 ± 0.20^**$$^
BUN (mg/dl)	24.2 ± 2.1	24.73 ± 1.6	26.3 ± 2.8	23.7 ± 1.3
Adipose tissue parameters				
Whole-body fat (g)	1.36 ± 0.05	3.33 ± 0.77^***^	4.28 ± 0.40^***$$^	4.34 ± 0.57^***$$^
Visceral fat area (cm^2^)	0.14 ± 0.03	0.47 ± 0.10^***^	0.72 ± 0.10^***$$$^	0.75 ± 0.09^***$$$^
Dorsal muscle density	0.92 ± 0.02	0.92 ± 0.03	0.88 ± 0.04	0.84 ± 0.03^***$$$^
Liver density	0.94 ± 0.02	0.94 ± 0.02	0.87 ± 0.04^***$$$^	0.86 ± 0.04^***$$$^
Adipocyte size (μm^2^)	1187 ± 635	3586 ± 1299^***^	5787 ± 1471^***$$$^	6137 ± 1717^***$$$#^
Adipocyte number	742 ± 156	547 ± 113^**^	416 ± 45^***^	382 ± 60^***$^

Results are mean ± SD values; *n* = 8 mice per group (Age 16 weeks).

The differences between groups were assessed by ANOVA followed by Tukey’s multiple comparisons test. The significance level is indicated as: ^*^p < 0.05; ^**^p < 0.01; ^***^p < 0.001 versus *Aqp7*^+/+^; ^$^*p* < 0.05; ^$$^*p* < 0.01; ^$$$^*p* < 0.001 versus *Aqp7*^+/+^ Glucose; ^#^*p* < 0.05; ^##^*p* < 0.01; ^###^*p* < 0.001 versus *Aqp7*^+/-^ Glucose.

*BUN* blood urea nitrogen.

These data indicate that AQP7 deficiency affects body weight and fat accumulation and induces adipocyte hypertrophy both at baseline and after glucose exposure.

### AQP7 deficiency drives adipose tissue remodeling and alters peritoneal membrane function

**Fig. 4:**
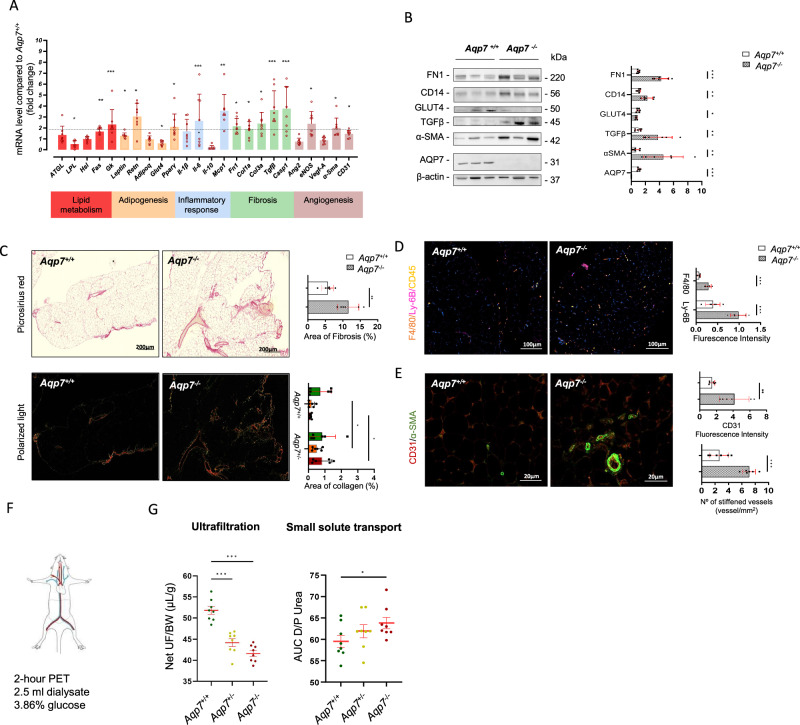
Effect of AQP7 deficiency on adipose tissue remodeling and function. **A** Transcript levels in the VAT, assessed by RT-qPCR, show enhanced lipogenesis and impaired lipid clearance with increased triglyceride synthesis, elevated expression of pro-inflammatory markers, and upregulation of genes involved in fibrosis, tissue remodeling, and vascular changes. These molecular changes were validated at **B** the protein level, confirming increased VAT remodeling in *Aqp7*^*−/−*^ mice. **C**–**E** Histological analysis reveal **C** *Aqp7*^*−/−*^ mice show a significant increase in VAT fibrosis, with an accumulation of orange and red fibers under polarized light. This is accompanied by elevated expression of **D** pro-inflammatory cytokines and chemokines, consistent with chronic low-grade inflammation, as shown by a stronger signal for F4/80 (macrophage marker) and Ly-6B (neutrophil marker) in the VAT of *Aqp7*^*−/−*^ mice. **E** *Aqp7*^*−/−*^ VAT shows increased CD31 expression, indicating increased capillary density, along with a greater number of stiffened vessels characterized by α-SMA rings, suggesting enhanced angiogenesis and vascular remodeling. **F**, **G** To assess the functional impact of VAT remodeling, peritoneal transport is evaluated using a well-established **F** mouse model of peritoneal dialysis. The peritoneal equilibration test **G** shows that *Aqp7*^*−/−*^ mice exhibit a significant reduction in water transport and increased small solutes transport across the membrane, indicating impaired peritoneal membrane function. Significant differences between groups were analysed using a *t*-test (**A**–**E**) or ANOVA followed by Tukey’s multiple comparisons test (**F**, **G**): *p < 0.05, **p < 0.01, ***p < 0.001.

In response to excessive fat accumulation, the adipose tissue undergoes significant alterations in structure, cellular composition, and secretory profile, which contributes to metabolic disturbances and chronic low-grade inflammation^24^. We evaluated the impact of AQP7 deletion on adipocyte remodeling and dysfunction in the VAT at baseline (Fig. 4).

Compared to *Aqp7*^+/+^ littermates, the VAT of *Aqp7*^−/−^ mice showed significantly altered mRNA levels of genes involved in various biological processes. Lipolysis-related markers, including hormone-sensitive lipase (HSL, *Lipe*), remained unchanged, while lipoprotein lipase (*Lpl*) was reduced. In contrast, enzymes involved in lipogenesis, such as fatty acid synthase (*Fasn*), and glycerol metabolism, like glycerol kinase (*Gk*), were significantly upregulated. There was also upregulation of pro-inflammatory markers such as leptin (*Lep*), resistin (*Retn*), MCP-1 (*Ccl2)*, and IL-6 (*Il-6*), as well as genes associated with fibrosis and tissue remodeling, including fibronectin (*Fn1*), collagen type I and type III alpha 1 (*Col1a*, *Col3a*), transforming growth factor beta (*Tgfβ*1), and caspase 1 (*Casp1*). Lastly, increased expression was observed for endothelial and vascular markers, including endothelial nitric oxide synthase (*Nos3*), CD31 (*Pecam-1*), and alpha-smooth muscle actin (*Acta2*) (Fig. 4A). Some of these changes were validated at the protein level (Fig. 4B). Together, these data suggest that AQP7 deletion promotes a significant remodeling of the VAT.

We next investigated whether the changes associated with deletion of AQP7 are reflected by morphological characteristics of the VAT in mice. Quantification of picrosirius red staining revealed that deletion of AQP7 significantly increased fibrosis in the VAT, with accumulation of orange and red fibers under polarized light reflecting fiber enlargement (Fig. 4C). The increased expression of pro-inflammatory cytokines and chemokines, supporting the development of chronic low-grade inflammation^24^, was verified by immunofluorescence. Increased signal for F4/80, a macrophage marker, and Ly-6B, a marker of neutrophils, was observed in the visceral peritoneum of the *Aqp7*^*−/−*^ mice compared to the wild-type controls (Fig. 4D). When compared to *Aqp7*^*+/+*^ mice, the VAT of the *Aqp7*^*−/−*^ mice showed a higher intensity of CD31 marking the capillaries and an increased number of stiffened vessels, characterized by a ring of α-SMA (Fig. 4E) suggesting enhanced angiogenesis and vasculopathy.

Since the VAT is a key component of the visceral peritoneum, inflammation may impair the function of the peritoneal membrane. To explore the functional impact of the VAT remodeling, we measured transport processes across the visceral peritoneum, using a well-established mouse model of PD (Fig. 4F and Table 3). The peritoneal equilibration test (PET) revealed that mice lacking AQP7 exhibited a significant reduction in water transport, assessed as ultrafiltration, associated with a significant increase in transport for small solutes across the membrane (Fig. 4G). These changes in transport were further evidenced in the *Aqp7* mice exposed to high glucose diet, characterized by higher levels of adipocytes enlargement and local and systemic inflammation (Supplementary Fig. 3), and a significant decrease in the ultrafiltration and increased small solute transport (Table 3).

**Table 3:** Impact of AQP7 deficiency and HGD on peritoneal transport parameters in the mouse

Groups	Genotype	BW (g)	NetUF/BW (μL/g)	NetUF (μL)	AUC D/P Urea
Baseline	*Aqp7*^+/+^	22.9 ± 1.0	55.0 ± 4.5	1259 ± 92	59.5 ± 4.6
	*Aqp7*^+/−^	25.4 ± 0.5^$$$^	44.5 ± 2.8^$$$^	1133 ± 96^$^	61.9 ± 4.3
	*Aqp7*^−/−^	26.3 ± 0.9^$$$^	41.4 ± 2.7^$$$^	1090 ± 73^$$^	63.7 ± 3.5^$^
Intervention: Control	*Aqp7*^+/+^	22.7 ± 0.5	48.8 ± 1.3	1139 ± 68	56.0 ± 1.5
Intervention: HGD	*Aqp7*^+/+^	25.1 ± 0.9**	38.7 ± 8.8***	968 ± 213*	59.5 ± 4.6
	*Aqp7*^+/−^	26.8 ± 1.0***^###^	33.5 ± 1.3***^###^	930 ± 67***^#^	61.9 ± 4.3**
	*Aqp7*^−/−^	26.7 ± 0.7***^##^	34.1 ± 3.6***^##^	907 ± 86***^##^	63.7 ± 3.5***^#^

Results are mean ± SD values; *n* =8 mice per group.

*BW* body weight, *UF* ultrafiltration, *AUC D/P* urea, area under the curve for dialysate to plasma concentration of urea.

$  *p <* 0.05; $$ *p* < 0.01; $$$ *p* < 0.001 to *Aqp7*^+/+^; ***p < 0.001 to Intervention control ; # *p* < 0.05 ;## *p* < 0.01 ; ### *p* < 0.001 to Intervention HGD *Aqp7*^+/+^.

Together, these data demonstrate that the functional loss of AQP7 leads to adipose tissue remodeling paralleled by disrupted metabolic processes, inflammation, and impaired functionality of the peritoneal membrane.

### Lack of AQP7 disrupts lipid metabolism and adipokine secretion during adipocyte maturation

Adipogenesis is a multistage process that involves a coordinated integration of multiple signaling pathways and transcription factors. Alterations of these mechanisms may lead to an impaired differentiation of adipocytes, which in turn may impact on their role as endocrine regulators^25^. Previous studies showed that AQP7 levels become only detectable 3 days after the induction of adipocyte differentiation, compatible with a role in adipogenesis^26^. To test this hypothesis, we used mouse embryonic fibroblasts (MEFs)—a well-established system to investigate the differentiation of adipocytes^27^.

We harvested MEFs from *Aqp7*^*−/−*^ and *Aqp7*^*+/+*^ littermates and induced controlled adipogenesis of these cells in vitro (Supplementary Fig. 4). MEFs exposure to an adipogenic induction medium activates key transcription factors, including peroxisome proliferator-activated receptor gamma (PPARγ) and CCAAT/enhancer-binding protein alpha (C/EBPα), which are crucial for the commitment to the adipocyte lineage. Then, between day 3 and day 7, the MEFs start to acquire a preadipocyte phenotype with morphological changes and formation of lipid droplets. In parallel, the expression and activation of PPARγ and C/EBPα increase, promoting the expression of genes involved in adipogenesis and lipid metabolism, together with lipid accumulation. From days 10 to 12, the adipocytes develop a fully functional lipid metabolism machinery and start to secrete adipokines.

**Fig. 5:**
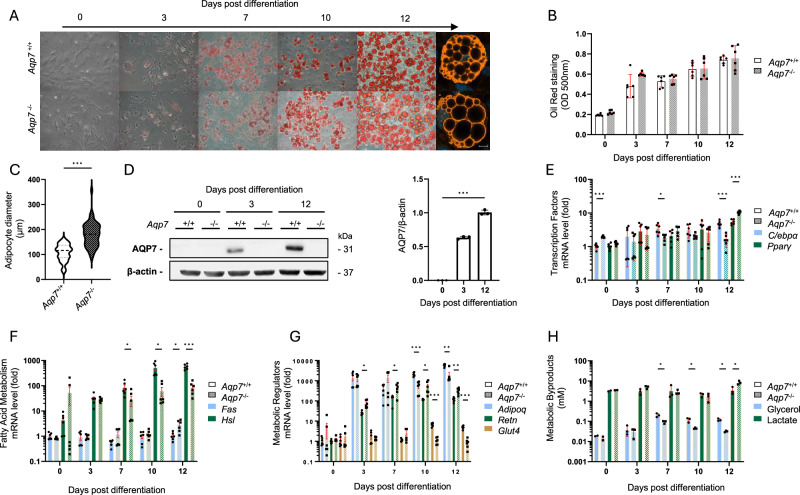
Effects of AQP7 deficiency on adipogenesis and adipocyte function. **A** Adipogenesis of the MEFs was assessed using oil red staining and fluorescence, over a period of 12 days to assess lipid accumulation and morphology. **B** No differences in lipid accumulation are observed between genotypes by the end of adipogenesis. **C** Adipocytes derived from *Aqp7*^*−/−*^ MEFs are significantly larger compared to those from *Aqp7*^*+/+*^ mice. **D** The expression of AQP7 begins on day 3 and progressively increases as adipocytes mature. **E** *Aqp7* deficiency leads to significant changes in the expression of transcription factors C/ebpa and Pparγ. **F** *Aqp7*^*−/−*^ adipocytes show altered lipid enzyme expression, including a marked decrease in hormone-sensitive lipase (HSL), which is involved in lipolysis. **G** *Aqp7*^*−/−*^ adipocytes exhibit a disrupted balance of adipokine expression, with decreased adiponectin (Adipoq) and increased resistin (Retn), along with defective induction of glucose transporter 4 (GLUT4). **H** Functionally, *Aqp7*^*−/−*^ adipocytes show a significant decrease in their capacity to release glycerol, with elevated lactate levels in the supernatant. Significant differences between groups were assessed by *t*-test (**C**), where the differences due to time points were assessed by one-way ANOVA (**D**) and the differences between genotype, time point or their interactions, were determined by two-way ANOVA (**B**, **E**–**H**): *p < 0.05, **p < 0.01, ***p < 0.001.

Over time (0 to 12 days), the adipogenesis of the MEFs was assessed using Oil Red O staining and fluorescence (Fig. 5A), with no differences between the two genotypes (Fig. 5B). However, at the end of adipogenesis, the adipocytes differentiated from *Aqp7*^−/−^ MEFs were significantly larger when compared to *Aqp7*^+/+^(Fig. 5C). The expression of AQP7 started at day 3 and progressively increased over adipocyte maturation (Fig. 5D). The AQP7 deficiency led to significant changes in the expression of the transcription factors *C/ebpa* and *Pparγ* (Fig. 5E) and some enzymes involved in lipid metabolism, FAS and HSL (Fig. 5F). In particular, the *Aqp7*^*−/−*^ adipocytes showed a major decrease in the expression of *Hsl*, involved in the breakdown of stored triglycerides into fatty acids and glycerol through the process of lipolysis (Fig. 5F). The profound defects of the *Aqp7*^*−/−*^ adipocytes were further evidenced by a major change in the balance of adipokines, with decreased mRNA levels of adiponectin (*Adipoq*, anti-inflammatory) contrasting with increased levels of resistin (*Retn*, pro-inflammatory), and a defective induction of the glucose transporter 4 (GLUT4) which plays a crucial role in glucose uptake and metabolism (Fig. 5G). Functionally, the *Aqp7*^*−/−*^ adipocytes showed a significant decrease of their capacity to release glycerol (Fig. 5H) paralleled by increased levels of lactate in the supernatant, suggesting an increased reliance on anaerobic glycolysis^28^.

These data show that AQP7 plays a major role in the terminal differentiation of adipocytes, impacting their function, metabolic state, and capacity to regulate local inflammation.

### AQP7 modulates the adipocyte secretome to regulate metabolic homeostasis

The diverse array of proteins, lipids, and signaling molecules—the secretome—released by adipocytes influences both local and systemic environments during adipogenesis^29^. To better understand the role of AQP7 on adipocyte biology and the potential systemic implications, we investigated the secretome during adipogenesis from both *Aqp7*^+/+^ and *Aqp7*^−/−^ MEFs (Supplementary Fig. 5).

**Fig. 6:**
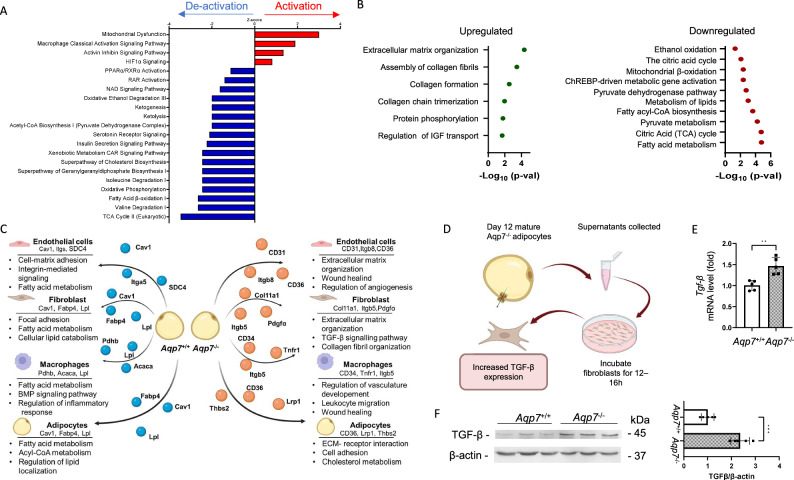
Impact of AQP7 deficiency on adipocyte secretory profile and pathways involved in adipocyte maturation and function. **A** Ingenuity pathway analysis (IPA) (Fisher’s exact test *p* < 0.05) reveals that *Aqp7*^−/−^ adipocytes enriched and activated pathways are related to mitochondrial dysfunction, hypoxia, and inflammatory responses, while pathways associated with metabolism and insulin signaling are significantly deactivated. The z-score predicts activation of pathways based on relationships and the direction of change of proteins in the dataset. Red: predicted activation, blue: predicted deactivation. **B** Twelve days post-adipogenesis induction, *Aqp7*^−/−^ adipocytes show alterations in the secretion of proteins, leading to enrichment of pathways as revealed by Reactome pathway analysis (Fisher’s exact test, *p* < 0.05). (green: pathways enriched by upregulated proteins, red: pathways enriched by downregulated proteins). **C** The changes in the secretome propose a shift toward fibrosis, inflammation, and altered lipid metabolism in *Aqp7*^−/−^ adipocytes, contrasting with the healthy tissue remodeling and immune regulation seen in *Aqp7*^+/+^ adipocytes. **D** To confirm these findings, supernatants from day 12 adipocytes of *Aqp7* mice were added to wild-type fibroblasts. Fibroblasts exposed to supernatants from *Aqp7*^−/−^ adipocytes show a significant upregulation of TGF-β expression at both **E** mRNA and **F** protein levels. Significant differences between groups were assessed by *t*-test: *p < 0.05, **p < 0.01, ***p < 0.001.

We identified 1761 murine proteins, including 1302 classified as secreted based on UniProt annotations^30^ or their presence in extracellular vesicles according to vesiclepedia^31^. Pathway analysis (Fig. 6A) revealed that the most activated pathways in *Aqp7*^−/−^ adipocytes were related to mitochondrial dysfunction and activation of hypoxia and inflammatory response pathways. In contrast, pathways related to metabolism and insulin signaling were significantly deactivated in *Aqp7*^−/−^ adipocytes.

Analysis of the secreted proteins relevant to the maturation process revealed 30 proteins that are upregulated in the *Aqp7*^*−/−*^ specific secretory profile and 129 proteins that are downregulated (Supplementary Fig. 6A). Upregulated proteins enriched pathways in the *Aqp7*^*−/−*^ secretome including extracellular matrix (ECM) organization, e.g. collagen fibril assembly and collagen formation, pointing to increased tissue remodeling and a potential for fibrosis development with a greater deposition of ECM, which could be a response to tissue stress or injury. In contrast, critical pathways involved in energy metabolism were downregulated, including ethanol oxidation, citric acid cycle, and mitochondrial β-oxidation, which suggests impaired mitochondrial function and a reduction in overall energy production. Furthermore, the downregulation of ChREBP-driven metabolic gene activation points to disrupted carbohydrate metabolism. Together, these alterations reflect a broader metabolic shift that could have significant effects on both cellular and tissue function (Fig. 6B).

To better understand how these secreted proteins may interact with diverse cell types within the VAT, we conducted an analysis of the available receptors in endothelial cells, macrophages, fibroblasts, and adipocytes that could interact to the observed secretory profile. The comparison of *Aqp7*^+/+^ and *Aqp7*^−/−^ adipocyte secretomes reveals significant differences in their possible interactions with fibroblasts, macrophages, endothelial cells, and adipocytes. The *Aqp7*^+/+^ adipocytes support regular tissue remodeling, immune regulation, vascular health, and healthy adipocyte interactions with their microenvironment. In contrast, *Aqp7*^−/−^ adipocytes show upregulation of fibrosis, inflammation, altered endothelial function, and dysregulated adipocyte behaviour, with disruptions in lipid metabolism and tissue architecture. These differences emphasize the shift toward fibrosis, inflammation, and altered lipid metabolism in the *Aqp7*^−/−^ secretome, contrasting with the functions seen in *Aqp7*^+/+^ adipocytes and were confirmed in the VAT of *Aqp7*-deficient mice (Fig. 6C and Supplementary Fig. 6B, C). To validate these analyses, we collected supernatants from 12-day MEFs-derived adipocytes obtained from both *Aqp7*^+/+^ and *Aqp7*^−/−^ mice and tested their effect on wild-type fibroblasts (Fig. 6D). Fibroblasts exposed to supernatants derived from *Aqp7*^−/−^ adipocytes exhibited a significant upregulation of TGF-β expression at mRNA (Fig. 6E) and protein (Fig. 6F) levels, compared to those treated with *Aqp7*^+/+^ supernatant. These findings suggest that factors secreted by *Aqp7*^−/−^ adipocytes create a pro-fibrotic microenvironment, driving enhanced TGF-β production in surrounding cells, possibly contributing to adipose tissue dysfunction and fibrosis.

Collectively, these results substantiate the key role of AQP7 in adipocyte differentiation and function, important for extracellular matrix remodeling and inflammatory responses.

## Discussion

In this study, we combined human, mouse, and in vitro cellular models to characterize the role of AQP7 in adipose tissue regulation, metabolism, and obesity-related dysfunction. The correlation between reduced expression of AQP7 in the adipose tissue and increased BMI was observed in humans, supporting the link between AQP7 downregulation, obesity, and metabolic disturbances^32^. In *Aqp7*-deficient mice, the lack of AQP7 leads to increased body weight and visceral fat accumulation, with adipocyte hypertrophy and chronic inflammation, consistent with adipose tissue dysfunction^33^. While prior studies have described a role for AQP7 in lipolysis^34–36^, our findings provide novel mechanistic insights by showing that AQP7 deficiency disrupts adipogenesis and alters the extracellular matrix composition, driving systemic inflammation and metabolic dysregulation. This highlights a broader role for the AQP7 channel beyond glycerol transport, positioning it as a critical regulator of adipose tissue remodeling and homeostasis.

The link between methylation of the *AQP7* locus and expression levels of *AQP7* in the adipose tissue aligns with prior research indicating that genetic and epigenetic variations in the *AQP7* promoter region impact obesity and related metabolic disorders, including type 2 diabetes mellitus susceptibility in humans^6–8^. This supports the emerging view that epigenetic regulation of aquaporins may serve as a molecular interface between environmental factors and metabolic health^37,38^.

Our studies in the *Aqp7* mouse model show a dose-dependent increase in body weight, hyperglycemia, hyperinsulinemia, and increased fat accumulation - particularly in the VAT. The increased ectopic fat deposition observed in the liver and skeletal muscle of *Aqp7*^−/−^ mice may arise from impaired lipolysis and reduced glycerol efflux in adipose tissue. The latter likely leads to intracellular triglyceride accumulation and adipocyte hypertrophy, which diminishes lipid mobilization capacity and causes excess lipids to be redirected toward non-adipose tissues^39^. These findings complement earlier studies^5^, while providing new insights into the metabolic consequences of AQP7 deficiency under high-glucose dietary stress. Aquaporin-7 thus appears to be critical not only for maintaining lipid mobilization^40,41^, but also for protecting against diet-induced metabolic deterioration.

Aquaporin-7 deficiency triggers a clear metabolic shift within adipocytes, characterized by reduced lipolytic activity and increased expression of enzymes involved in lipogenesis and glycerol utilization. This imbalance favors lipid accumulation, contributing to adipocyte hypertrophy and overall VAT expansion. In parallel, we demonstrate that AQP7 deficiency promotes significant remodeling of VAT, with increased ECM accumulation, immune cell infiltration and vascular alterations. Specifically, we observed upregulation of fibrotic markers, indicating excessive ECM accumulation that contributes to tissue stiffening. This fibrotic remodeling likely impairs the diffusion of oxygen and nutrients, leading to localized hypoxia—a key driver of VAT dysfunction^42^. In parallel, AQP7-deficient adipose tissue showed increased levels of inflammatory markers such as MCP-1 and IL-6, supporting the presence of chronic low-grade inflammation^43^. Hypoxic and inflammatory conditions stimulate the secretion of proangiogenic factors, including VEGF, promoting neovascularization as a compensatory response^44^. However, rather than restoring normal tissue architecture, these new vessels often undergo structural remodeling, including vascular wall thickening due to smooth muscle cell proliferation to adapt to the mechanical and metabolic demands of adipose tissue expansion^45^. These structural changes—fibrosis, inflammation and pathological vascular remodeling—are hallmarks of adipose tissue dysfunction in obesity and metabolic disease^46,47^.

The VAT is particularly exposed to glucose in the context of PD, which implies daily contact to high-glucose dialysis solutions. Previous studies showed that this chronic exposure to glucose contributes to significant weight gain and visceral adiposity, closely linked to systemic inflammation and complications^48^. Here, we demonstrate that under caloric excess, the enlargement of adipocytes compromises peritoneal membrane function in *Aqp7*-deficient mice, with decreased water transport paralleled by increased small solute transport, likely reflecting the pro-inflammatory state driven by adipocyte hypertrophy^14^. To our knowledge, this is the first study to link AQP7 deficiency to structural and inflammatory changes in VAT that impair peritoneal membrane function, building upon our previous finding that adipocyte size/volume affects ultrafiltration capacity during fasting^10^.

The role of AQP7 in adipocyte differentiation was further investigated using MEFs obtained from the *Aqp7* mice. These studies demonstrated that although AQP7 is not required for adipocyte differentiation, its absence leads to adipocyte hypertrophy, a pro-inflammatory secretory profile, and a shift toward increased lipid storage and impaired breakdown—hallmarks of hypertrophy and metabolic dysfunction. The observed imbalance between *C/ebpα* and *Pparγ*, along with downregulation of *Lipe* and *Slc2a4*, suggests that AQP7 acts upstream of key regulators of lipid metabolism and insulin sensitivity, disrupting lipid metabolism and adipocyte function^49–51^. Secretome analyses corroborate these finding,s showing significant alterations in the extracellular matrix and metabolic pathways in *Aqp7*^−/−^ adipocytes. The observed shift toward increased fibrosis and inflammation supports a role of AQP7 in modulating intercellular signaling within the adipose tissue, with functional evidence that secreted factors from *Aqp7*^−/−^ adipocytes promote fibroblast activation. These findings provide new mechanistic insights into how AQP7 loss contributes to adipose tissue dysfunction and systemic metabolic disturbances.

While previous research has largely focused on the role of AQP7 in glycerol transport^4,41,52^, our multi-level evidence findings point to a multifaceted involvement of AQP7 in adipocyte hypertrophy, ECM remodeling, inflammation, and metabolic dysfunction. This positions AQP7 not only as a metabolic facilitator but as an important regulator of adipose tissue integrity and inter-organ communication. Limitations of the current studies include the relatively small size of the human cohort, limiting the statistical power of methylation-expression correlations; the use of mouse models, which may not fully reflect human physiology and metabolism; and the focus on AQP7, which may have overlooked other regulatory mechanisms and signaling pathways that also affect adipose tissue function^53,54^. Lastly, the direct causality of some of the pathways has not been investigated. The specific implications of AQP7 modulation for the adipose tissue and metabolic health will require further investigations, including AQP7 modulation in metabolic disease contexts.

In conclusion, our data support that AQP7 plays a crucial role in adipose tissue homeostasis and metabolic resilience, especially under chronic glucose exposure. Targeting AQP7 may offer novel therapeutic possibilities to combat obesity-related complications, particularly in chronic high-glucose exposure settings such as PD.

## Methods

### CpG methylation in human adipose tissue

CpG methylation and microarray gene expression data for the *AQP7* locus were extracted from a previously published cohort examining the global CpG methylation profile in subcutaneous white adipose tissue of women spanning a range of BMI^55^. From this cohort, 65 women were included where *AQP7* CpG methylation and gene expression were available. Briefly, an abdominal subcutaneous white adipose tissue biopsy was obtained by needle aspiration at 8:00 AM after an overnight fast. Gene expression was performed as described on whole white adipose tissue pieces using the Gene 1.0 or 1.1 ST Affymetrix arrays (Affymetrix, Inc., Santa Clara, CA)^56^. For global CpG methylation, the adipocytes were first isolated using collagenase digestion before DNA isolation. The Infinium Human Methylation EPIC BeadChip (Illumina, San Diego, CA) was used for CpG profiling^55^. Obesity was defined as a BMI > 30, while a lean/healthy BMI is considered < 25, according to the WHO^57^. The study was approved by the regional ethics board in Stockholm, and written informed consent was obtained from each subject.

### Experimental animals and a high glucose diet

Knock-out (*Aqp7*^−/−^), haplodeficient (*Aqp7*^*+/−*^) and wild-type (*Aqp7*^+/+^) littermates (C57BL/6J background; aged 6 to 8 weeks)^10,58^ were used to analyse the effect of varying AQP7 expression in a glucose-induced obesity model^59^. The mice were randomized into two groups: one group received water supplemented with 30% (vol/vol) dextrose, while the other group received plain water without any supplementation for 8 weeks (Supplementary Fig. 1A). All the mice were allowed free access to a standard laboratory diet (Scientific animal food & engineering, Augy, France). Food and water intake, as well as body weight, were measured once per week. All mice were housed in an air-conditioned room with a 12/12-h dark-light cycle. The experiments were conducted in accordance with the National Research Council Guide for the Care and Use of Laboratory Animals and approved by the ethics committee of the UCLouvain Medical School (Brussels, Belgium).

### Micro-computed tomography analysis

Micro-CT scans were performed on mice anesthetized with isoflurane/oxygen to monitor changes in body composition and fat mass. Scanning was performed with Skyscan 1278 (Bruker, Kontich, Belgium) at 50 µm voxel resolution using a source voltage of 65 kV and a current of 770 uA as described^60^. Raw images were then reconstructed with an isotropic voxel size of (51 × 51 × 51) µm³.

Whole-body composition analysis in mice was performed using micro-CT to quantify adipose tissue, lean tissue, and skeletal volumes. Visceral fat area was assessed at the L4 level via single-slice analysis, using semi-automatic region-of-interest (ROI) delineation and a grey value threshold of 30–57. Dorsal muscle analysis involved manual ROI selection at L4 and L5, applying a grey value threshold (30–97) to exclude non-lean tissue, with muscle density subsequently measured. Virtual liver biopsy was conducted by placing a 3D cylindrical ROI in the liver, and the mean density (HU) was automatically computed. Dorsal muscle and liver densities were normalized to spleen density as an internal control (Supplementary Fig. 1B). All analyses were performed using SkyScan software (CT Analyser version 1.17.7.2).

### Peritoneal transport studies, blood and tissue sampling

A peritoneal equilibration test (PET) with 2.5 mL of 3.86% glucose dialysate was performed to evaluate peritoneal transport parameters, as previously described^61^. Briefly, the mice underwent a 2-h PET following the administration of 2.5 mL of 3.86% glucose dialysate. After the dwell, the dialysate was collected from the peritoneal cavity, and the net ultrafiltration (UF) volume was determined. Mice were euthanized using an overdose of sevoflurane, in accordance with approved ethical guidelines. Blood samples were collected from the inferior vena cava and immediately centrifuged to separate the serum. Serum was stored at −20 °C until analysis. Triglycerides and urea were assayed using Dri Chem NX500i (Fujifilm, Tokyo, Japan). The visceral peritoneum was snap-frozen in liquid nitrogen and stored at −80 °C, or routinely fixed in 4% paraformaldehyde in 0.1 M phosphate buffer (VWR, Brussels, Belgium). Protein concentration was assayed using the Pierce BCA protein assay kit (Thermo Fisher Scientific, Waltham, MA, USA).

### MEFs cell culture and differentiation

Mouse embryonic fibroblasts (MEFs) were prepared from 13.5 days *Aqp7*^+/+^ and *Aqp7*^−/−^ mouse embryos as previously described^27^. The embryos were extracted from the uterus and minced with a sterile scalpel under a cell culture hood. Single cell suspensions were prepared by incubating for 20 min at 37 °C with 0.25% trypsin/EDTA. The reaction was stopped with the addition of medium containing 10% fetal bovine serum (FBS). DNase was added at the final concentration of 100 μg/ml and incubated for 15 min at 37 °C. Cells were grown in 5% CO_2_ incubator at 37 °C in culture medium (high glucose DMEM) supplemented with 10% (v/v) FBS. Adipogenesis was induced in two days postconfluent cells using a well-established combination of proadipogenic factors: 1 μM dexamethasone, 0.5 mM 3-isobutyl-1-methylxanthine (IBMX), 5 μg/ml insulin, and 0.5 μM rosiglitazone in pre-differentiation medium (high glucose DMEM supplemented with 10% FBS). Cells were exposed to these factors for 2 days, following which the medium was substituted with post-differentiation medium (high glucose DMEM supplemented with 10% FBS) containing only 5 μg/ml insulin. Post-differentiation medium was renewed every other day until day 12. On day 12 post-differentiation, the supernatants from both *Aqp7*^+/+^ and *Aqp7*^−/−^ cell cultures were collected and used for further analysis. Wild-type fibroblasts were subsequently exposed to the 0.22-μm-filtered supernatants from both *Aqp7*^+/+^ and *Aqp7*^−/−^ cells for 12 h. After the exposure period, the cells were harvested and processed for downstream assays to evaluate the effects of the different conditioned media.

### Lipid visualization/quantification using Oil Red staining

After being washed twice with PBS, the differentiated cells were fixed for 15 min at room temperature using 4% paraformaldehyde (PFA) in PBS. Subsequently, the fixed cells underwent two washes with dH_2_O, one wash with 60% isopropanol, and were then stained for 5 min with 0.2% Oil Red O in 60% isopropanol. Excess stain was removed by washing three times with tap H_2_O. Images were captured using an optical phase contrast microscope fitted with a digital camera. Spectrophotometric evaluation of the dye (Abs 500 nm) was carried out after lipid extraction using 100% isopropyl alcohol for 10 min.

### Enzyme-linked immunosorbent assays

The levels of insulin (Mercodia, Uppsala, Sweden), MCP-1 (Sigma-Aldrich, St Louis, MO, USA), IL-6 and IL-1β (R&D Systems, Wiesbaden-Nordenstadt, Germany) in serum or dialysate samples were quantified in duplicate using Enzyme-linked immunosorbent assay, according to the manufacturer’s protocols.

### Glycerol, free fatty acid, and lactate analysis

Colorimetric assay kits were used to quantify glycerol (#MAK117, Sigma-Aldrich), free fatty acid (#MAK044-1KT, Sigma-Aldrich), and lactate (#MAK064, Sigma-Aldrich) released from adipocytes in serum or cell culture medium samples and performed as per the manufacturer’s instructions.

### RT-PCR and quantitative real-time RT-PCR

Total RNA from mouse visceral peritoneum was extracted with Trizol (Invitrogen, Merelbeke, Belgium) and using AurumTM Total RNA fatty and fibrous tissue kit (Bio-Rad, Hercules, CA, USA) according to the manufacturer’s protocol. DNase I treatment was performed to eliminate genomic DNA contamination. One microgram of RNA was used to perform the reverse-transcriptase reaction with iScriptTM cDNA Synthesis Kit (Bio-Rad). Three reference genes (*Gapdh*, *Actb*, and *36b4*) not influenced by treatment protocols were used for the normalization. The sequences and efficiencies of the primers are given in (Supplementary Table 1). Changes in the mRNA levels of target genes were determined by semiquantitative reverse-transcriptase-polymerase chain reaction (RT-PCR) with an iCycler IQ system (Bio-Rad, Hercules, CA, USA) using SYBR Green I detection as described previously^61^. The PCR conditions were 95°C for 3 min followed by 40 cycles of 30 s at 95 °C, 15 s at 60 °C and 1 min at 72 °C. The relative changes in target mRNA between groups were determined by using the relation 2^−ΔΔCt^.

### Tissue staining and immunofluorescence

Samples from the peritoneum were fixed in 4% paraformaldehyde (PAF), embedded in paraffin and cut into 5 µm sections. Hematoxylin-eosin (HE), picrosirius red staining and immunostaining were performed as previously described^10^. The areas of adipocytes in the visceral peritoneum (HE) were calculated (π∙radius²) in at least 50 cells per section, and the cells were automatically counted using ImageJ software. Sirius red staining was observed under polarized light microscopy (AXIOSCAN; Zeiss, Oberkochen, Germany). The area of collagen fibers as a function of their color was quantified from tissue sections stained^62^. The color corresponds to relative fiber thickness from thin green fibers to increasingly thick yellow, orange, and red fibers.

For immunofluorescence, the tissue sections were deparaffinized and rehydrated using a series of ethanol dilutions of decreasing concentration. Antigen retrieval was performed using sodium citrate buffer (10 mM, in distilled water, pH 6.0). The tissue sections were heated in the microwave at 900 W for 4 min, followed by 90 W for 15 min, then 900 W for 1 min 30 s and finally cooled at room temperature for 10 min. The slides were then blocked with blocking buffer (1% BSA, 0.2% non-fat dry milk in PBS) for 60 min and incubated with primary antibody in blocking buffer overnight at 4 °C. After three washes in 0.1% Tween 20 (v/v in PBS), the tissue slides were incubated with fluorophore-conjugated secondary antibody (Life Technologies, Carlsbad, CA, USA) diluted in blocking buffer at room temperature for 1 h. The complete list of antibodies used for immunofluorescence is included in Supplementary Table 2. Finally, the slides were mounted in Prolong Gold anti-fade reagent (Invitrogen, Waltham, MA, USA) and examined using a Zeiss LSM 510 Meta Confocal microscope (Carl Zeiss, Jena, Germany).

### Proteomics analysis of cell culture supernatants (secretome analysis)

#### Sample preparation

Following experimental treatments, supernatants were collected and frozen until analysis at −80 °C. Combinatorial peptide ligand library (CPLL) equalizer beads (ProteoMiner, Bio-Rad, Hercules, CA, USA) were prepared per manufacturer’s protocol. About 3 mL supernatant were mixed with 600 μl CPLL bead solution (150 μl bead bed volume) and incubated on a roller mixer (overnight, 4 °C). Total protein concentration was determined (Pierce 660 nm Protein Assay, Thermo Fisher Scientific) per the manufacturer’s manual. Pure FBS samples were processed in the same way as the samples in order to generate an exclusion list for bovine proteins contained in the supernatant.

#### Protein digestion and labelling

About 110 µg of each sample were used. Before digestion, the pH was adjusted to neutral. Digestion was performed using single-pot, solid-phase enhanced sample preparation (SP3). Briefly, the reduced (10 mM DTT for 1 h at 56 °C) and alkylated (55 mM IAA, 30 min at RT) proteins were bound to SP3 beads (10:1 beads:protein ratio, GE Healthcare, Illinois, USA), washed with 80% ethanol and acetonitrile, and subjected to on-bead digestion with trypsin/LysC (1:25 protease:protein ratio, Promega, Madison, Wisconsin, USA) overnight at 37 °C in 50 mM ammonium bicarbonate, pH 8.5 (Sigma-Aldrich, Schnelldorf, Germany). Following elution and desalting (Peptide Desalting columns; Thermo Fisher Scientific), peptides were dried in a vacuum concentrator. Peptide concentration was determined (Colorimetric Peptide Assay, Thermo Fisher Scientific) according to the manufacturer’s protocol. An internal pooled standard was prepared of equal parts of all samples.

Peptides were labeled with isobaric mass tags for multiplexing (TMTpro, Thermo Fisher Scientific) according to the instructions provided by the manufacturer, with minor modifications. TMTpro reagents were reconstituted with acetonitrile and 70 µg each sample were labeled with 210 µg of TMTpro reagent. After incubation for 1 h at RT the reaction was quenched by the addition of 5% hydroxylamine (Sigma-Aldrich) in TEAB for 15 min at RT. Labeling efficiency was determined by LC-MS.

#### Offline fractionation

Pooled labeled samples were concentrated and desalted (Pierce Peptide Desalting Columns; Thermo Fisher Scientific). Eluates were dried in a vacuum concentrator and reconstituted (20 mM ammonia formate, pH 10) prior to fractionation at basic pH. All fractionations were performed using an UltiMate 3000 system (Thermo Fisher Scientific). Peptides were separated on a Gemini-NX C18 (150 × 2 mm, 3 µm, 110 A, Phenomenex, Torrance, USA) column in 20 mM ammonia formate buffer, pH 10.

#### Liquid chromatography–mass spectrometry (LC-MS)

Fractions were analyzed on an Ultimate 3000 RSLC nano coupled directly to an Exploris 480 with FAIMSpro (all Thermo Fisher Scientific). Samples were injected onto a reversed-phase C18 column (50 cm×75 µm i.d., packed in-house) and eluted with a gradient of 4 to 38% mobile phase B over 94 min by applying a flow rate of 230 nl/min. MS scans were performed in the range from m/z 375–1650 at a resolution of 120,000 (at m/z = 200). MS/MS scans were performed, choosing a resolution of 30,000 with the turboTMT mode for TMpro Reagent; normalized collision energy of 33%; isolation width of 0.7 m/z and dynamic exclusion of 90 s. Two different FAIMS voltages were applied (−40 and −60 V) with a cycle time of 1.5 s per voltage. FAIMS was operated in standard resolution mode with a static carrier gas flow of 4.6 L/min.

#### Spectral analysis

The acquired raw MS data files were processed and analyzed using ProteomeDiscoverer (v2.4.0.305, Thermo Fisher). SequestHT was used as a search engine, and the following parameters were chosen: database: *Mus musculus* (SwissProt, downloaded on 2022-06-15) as well as the bovine fasta file created from the analysis of FCS; enzyme: trypsin; max. missed cleavage sites: 2; static modifications: TMTpro (K and peptide N-terminus) and carbamidomethyl (C); dynamic modifications: oxidation (M), acetyl (protein N-terminus), Met-loss (M) and Met-loss + acetyl (M); precursor mass tolerance: 10 ppm; fragment mass tolerance: 0.02 Da. For reporter ion quantification, the most intense m/z in a 20 ppm window around the theoretical m/z was used. Correction of isotopic impurities for reporter ion intensities was applied. Only unique peptides, above an average S/N threshold of 10, were used for quantification. Normalization was based on total peptide amount and scaling mode on the control average (internal standard) in the first round. All Master Proteins and Master Protein candidates with FDR <0.01 were exported, and the assignment to a species was checked. A fasta file was created out of all mouse proteins with more than one matching peptide and used in a second analysis for normalization of the data. Afterwards, all Master Proteins and Master Protein candidates with FDR <0.01 were exported, and the assignment to a species was checked. Single peptide IDs were excluded from the dataset, and only peptides and proteins with FDR <0.01 were reported.

#### Data analysis

Results are expressed as mean ± SD. Differences between groups were assessed by unpaired *t*-test, one-way ANOVA followed by Tukey’s multiple comparisons test or two-way ANOVA (to assess genotype effect in different time points), using GraphPad Prism Software version 9.4.0 (San Diego, California, USA). A *p* value <0.05 was considered statistically significant.

Statistical analyses of the proteomics data and graphical representations of results were performed using R (v4.0.3; http://www.r-project.org/). Ingenuity Pathway Analysis (IPA 7.0, Qiagen, http://www.ingenuity.com) or Reactome Pathway Analysis (https://reactome.org) were used to identify involvement of pathways, their respective predicted activation or deactivation patterns, and their enrichment based on differentially abundant proteins (one-tailed Fisher’s exact test, alpha 0.05). The IPA-calculated z-score assessed the activation state based on observed and predicted up/downregulation patterns, while Reactome pathway analysis provided insights into the biological relevance of the dataset by mapping proteins to known curated pathways. Differential protein abundances were analyzed with linear models for microarray data (LIMMA) using the R package “limma” as described before^63^. The Human Protein Atlas^64^ was used to extract cell-specific proteins of the most common cell types in adipose tissue. These proteins were then filtered for receptors according to Browaeys et al^40^. For cell-specific patterns of communications, receptors present in all four cell types were removed from the analysis.

## Supplementary information

Supplementary Information

## Data Availability

All proteomics mass spectrometry data have been deposited into the ProteomeXchange Consortium (http://proteomecentral.proteomexchange.org) via the PRIDE partner repository with dataset identifiers PXD062085 and PXD062778.
