# Supplementary material for: AQP7 deficiency drives adipose tissue remodeling and disrupts homeostasis
Source: NPJ Metab Health Dis. 2025 Nov 7;3:44. doi: 10.1038/s44324-025-00085-y (PMC12595075; doi:10.1038/s44324-025-00085-y)

AQP7 deficiency drives adipose tissue remodeling and disrupts homeostasis

Supplementary appendix

Table of Contents

Supplementary Table 1: Primers Used for Quantitative RT-PCR Analysis.................... 2

Supplementary Table 2: Antibodies Used for immunofluorescence. 4

Supplementary Table 3: Correlation between CpG methylation in the *AQP7* locus, expression of AQP7 and body mass index (BMI). 5

Supplementary Figure 1: Experimental design and micro-CT analysis......................... 6

Supplementary Figure 2: DNA methylation analysis of *AQP7* Expression and BMI. 7

Supplementary Figure 3: Impact of AQP7 deficiency and glucose consumption on inflammation 9

Supplementary Figure 4: Timeline of adipogenesis.. 10

Supplementary Figure 5: Workflow of the secretome analysis 11

Supplementary Figure 6: Secretome Profiling of Adipocyte Maturation. 12

Supplementary Table 1: Primers Used for Quantitative RT-PCR Analysis.

Gene	Forward primers (5'-3')	Reverse primers (5'-3')	PCR products (bps)	Efficiency
Lipe	AACAGCCTGGCAAAATCTGAGGG	GCCCAGTTCTTGAGGTAGG	152	0.99 ± 0.04
Fasn	GATGACATCGTGCATGCCTTTGTG	CTGGGAGAGACAGCCATCT	150	1.02 ± 0.02
Gk	GCTACAAGCAGACATTCTGTA	CTTCATCACAGCTTTCTT CCA	220	1.01 ± 0.02
Lep	GGACTTCATTCCTGGGCTTC	TGGAGGAGGTCTCGGAGATT	147	1.01 ± 0.03
Retn	GTACCCACGGGATGAAGAACC	GCAGAGCCACAGGAGCAG	253	0.91 ± 0.01
Adipoq	AGATGGCACTCCTGGAGAGAA	TTCTCCAGGCTCTCCTTTCCT	145	0.99± 0.01
Slc2a4	GATTCTGCTGCCCTTCTGTC	CAGCTCAGCTAGTGCGTCAG	130	0.97± 0.01
Cebpα	GCAGTGTGCACGTCTATGCT	AGCCCACTTCATTTCATTGG	198	1.00 ± 0.02
Pparγ	CCCTGGCAAAGCATTTGTAT	GAAACTGGCACCCTTGAAAA	225	0.97 ± 0.01
Il-1β	AGATGAAGGGCTGCTTCCAAA	AATGAGTGATACTGCCTGCCTGA	113	0.98± 0.03
Il-6	GGAGCCCACCAAGAACGATA	AGCCTCCGACTTGTGAAGTG	199	0.98± 0.06
Il-10	CTGTTTCCATTGGGGACACT	AAGTGTGGCCAGCCTTAGAA	112	0.97 ± 0.05
Ccl2	TTCTCCAGGCTCTCCTTTCCT	GCTCACATCATGAGCTCCAA	159	1.00 ± 0.01
Fn1	GCAAGCCAGTTTCCATCAAT	CATTTTTGGGAGTGGTGGTC	150	0.95 ± 0.06
Col1a	GATCTCCTGGTGCTGATGGA	GACCTTGTTTGCCAGGTTCA	156	0.96 ± 0.06
Col3a	TCCTGGTGGTCCTGGTACT	TTGCCAGGAGAACCACTGTT	153	0.96 ± 0.01
Casp1	ACCCTCAAGTTTTGCCCTTT	GATCCTCCAGCAGCAACTTC	193	1.00 ±.03
Tgfβ1	TGCTTCAGCTCCACAGAGAA	TGGTTGTAGAGGGCAAGGAC	182	0.96 ± 0.04
Angpt2	CCAACTACAGGATTCACC TTACAGG	CATTGTCCGAATCCTTTGTGC	102	1.02 ± 0.03
Nos3	GTGTGAAGGCAACCATTC TGT	TCATACTCATCCATGCACAGG	121	0.94 ± 0.06
Vegf-A	ACCATGCAGATCATGCGGAT	GCGCTTTCGTTTTTGACCCT	159	0.99 ± 0.05
Actb	TGCCCATCTATGAGGGCTAC	CCCGTTCAGTCAGGATCTTC	102	0.99 ± 0.03
Acta2	CTGACAGAGGCACCACTGAA	CATCTCCAGAGTCCAGCACA	160	1.01 ± 0.02
Pecam1	AAACAGAAACCCGTGGAGATGTC	TCATCATAACCGTAATGGCTGTTG	110	0.98 ± 0.02
36B4	CTTCATTGTGGGAGCAGACA	TTCTCCAGAGCTGGGTTGTT	150	0.98 ± 0.03

Gapdh	TGCACCACCAACTGCTTAGC	GGATGCAGGGATGATGTTCT	176	1.04 ± 0.03
Pnpla2	CAACGCCACTCACATCTACGG	GGACACCTCAATAATGTTGGCAC	106	0.97 ± 0.05
Lpl	CTTTCACTCGGATCCTCTCG	AGGTGGACATCGGAGAACTG	117	1.02 ± 0.01
Ppargc1a	TCACACCAAACCCACAGAAA	CTTGGGGTCATTTGGTGACT	151	1.05 ± 0.03
mt-Nd1	TAGAACGCAAAATCTTAGG	TGCTAGTGTGAGTGATAGGG	179	0.98 ± 0.02
mt-Nd2	TCCGAGCATCTTATCCACG	GTATGGTGGTAACTCCCGCTG	155	1.01 ± 0.04
Cpt2	GTATGGTGGTAACTCCCGCTG	GCAGTGCTGCAGGATTCATA	183	1.02 ± 0.03
Drp1	CCGTGACAAATGAAATGGTG	CTGAAGGCAGCTCTCTTGCT	150	0.99 ± 0.04
Opa1	GGACCCAAGAGCAGGTGTT	TGGATCGACTTCCACTCCTC	191	1.02 ± 0.02
Hif1a	CCCAAAGACAATAGCTTCGCA	ACAGTCACCTGGTTGCTGCAA	112	1.02 ± 0.03
Slc2a1	ACCTCTTCCGAACCGACAGAT	TCTGGAGCCATCAAAGTCCTG	102	1.01 ± 0.01
Ldha	CAAAGTCCAAGATGGCAACCC	AGCACCAACCCCAACAACTGT	101	0.98 ± 0.03
Pdk1	CCAAGACCTCGTGTTGAGACC	AATACACGTCTCAGGTCTCCTTGG	132	0.98 ± 0.02

Supplementary Table 2: Antibodies Used for immunofluorescence.

Primary Antibody	Host	Dilution	Company	Secondary Antibody	Dilution	Company	Fluorochrome	Company
F4/80	Rabbit	1:200	Cell signaling, ref. 70076S	Anti-Rabbit	-	Dako ref. K4003	CF647	Invitrogen
Ly-6B	Rat	1:400	Abcam ref. AB53457	Rabbit-Anti-rat	1:200	Vector laboratories, ref. AI-4001	CF754	Invitrogen
CD45	Rabbit	1:100	CellSignaling ref. 70257S	Anti-Rabbit	-	Dako ref. K4003	CF555	Invitrogen
CD31	Goat	1:200	Santa Cruz ref. 1506	Anti-Goat	1:400	-	Alexas Fluor™ 568 Ref. A-11057	Thermo Fisher
A-SMA	Rabbit	1:400	Novus Biologicals ref. NB600-531	Anti-Rabbit	1:400	-	Alexas Fluor™ 488 Ref. A-21206	Thermo Ficher

Supplementary Table 3: Correlation between CpG methylation in the *AQP7* locus, expression of AQP7 and body mass index (BMI).

	**AQP7 Expression**		**BMI**	
**CpG site**	**Pearson's coefficient**	**p**	**Pearson's coefficient**	**p**
**cg16494465**	-0.2644	**0.033**	0.1268	0.3140
**cg15050672**	-0.3409	**0.005**	0.1021	0.4185
**cg02075232**	-0.4018	**0.0009**	0.3169	**0.0101**
**cg09773437**	-0.2442	**0.050**	0.1973	0.1153
**cg01836353**	-0.4005	**0.0009**	0.4478	**0.0002**
**cg15240092**	-0.3562	**0.0036**	0.1955	0.1187
**cg09705264**	-0.06297	0.6182	0.1477	0.2404
**cg16447312**	-0.4020	**0.0009**	0.4716	**<0.0001**
**cg08004206**	-0.06516	0.6061	0.05316	0.6741

Supplementary Figure 1: Experimental design and micro-CT analysis.

(A) Schematic representation of the 8-week study, in which mice are monitored from 8 to 16 weeks of age. Mice are assigned to either a control group or a glucose-supplemented water group. At the end of the study, metabolic phenotype, body composition, adipocyte size, and peritoneal transport are analysed. **(B)** Illustration of micro-CT scanning methodology used to evaluate body composition, measure visceral fat area, and calculate the fat infiltration index in muscle and liver.

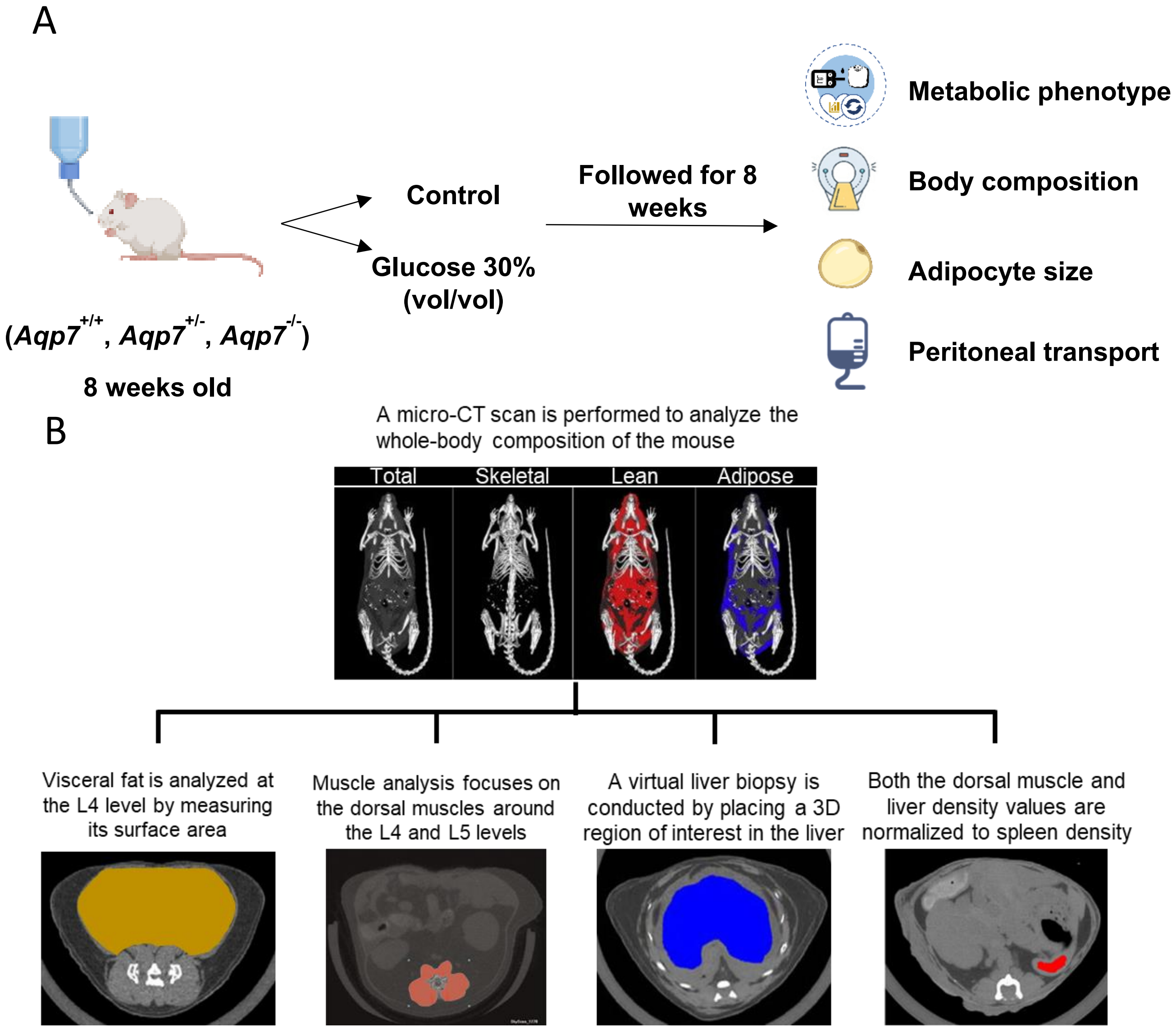

Supplementary Figure 2: DNA methylation analysis of *AQP7* Expression and BMI.

(A) DNA methylation analysis identifies seven sites that negatively correlate with *AQP7* expression in subcutaneous white adipose tissue. **(B)** Three sites exhibit a positive correlation with BMI, indicating a potential association between DNA methylation patterns, *AQP7* expression, and obesity-related traits.

A

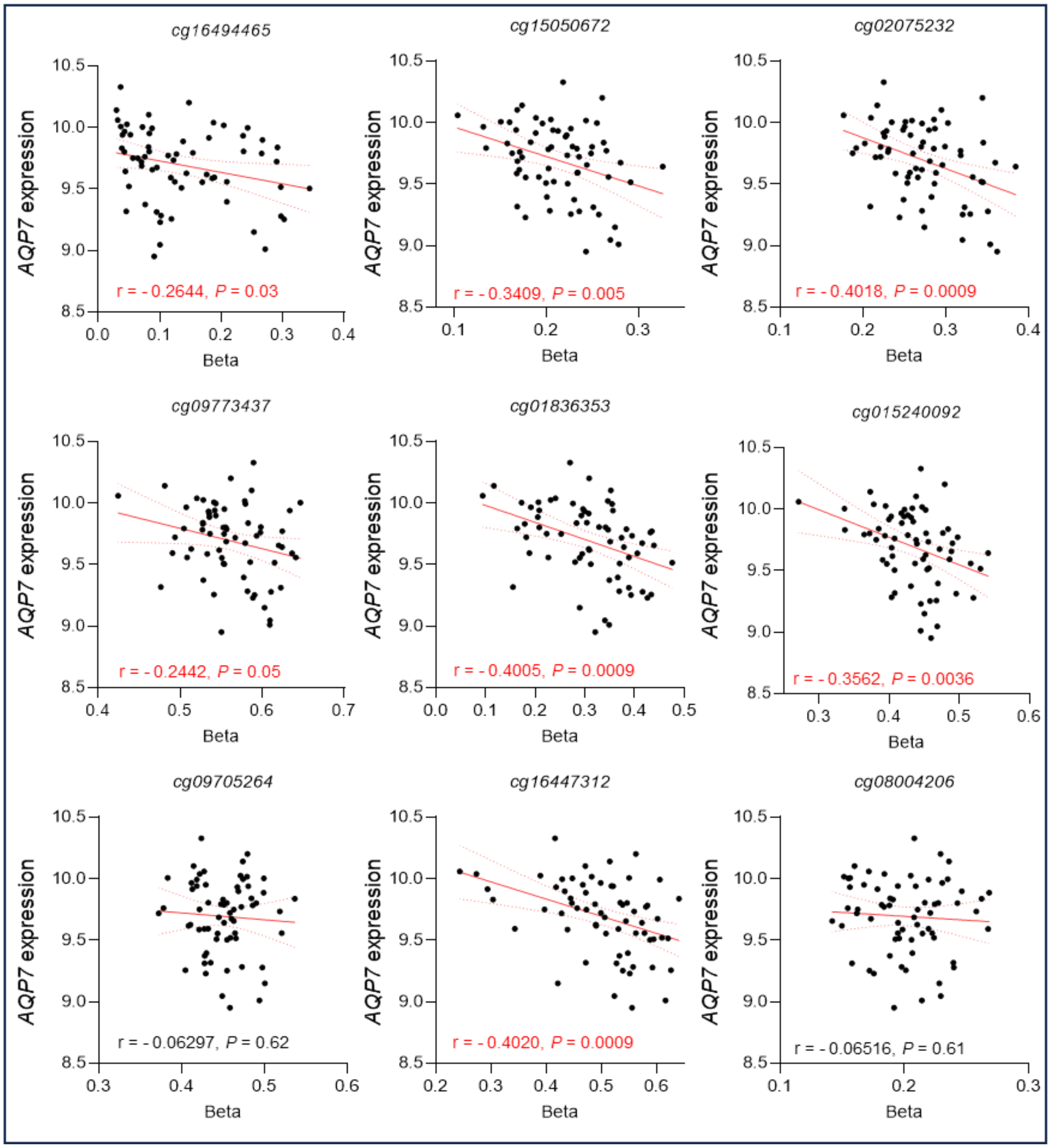

B

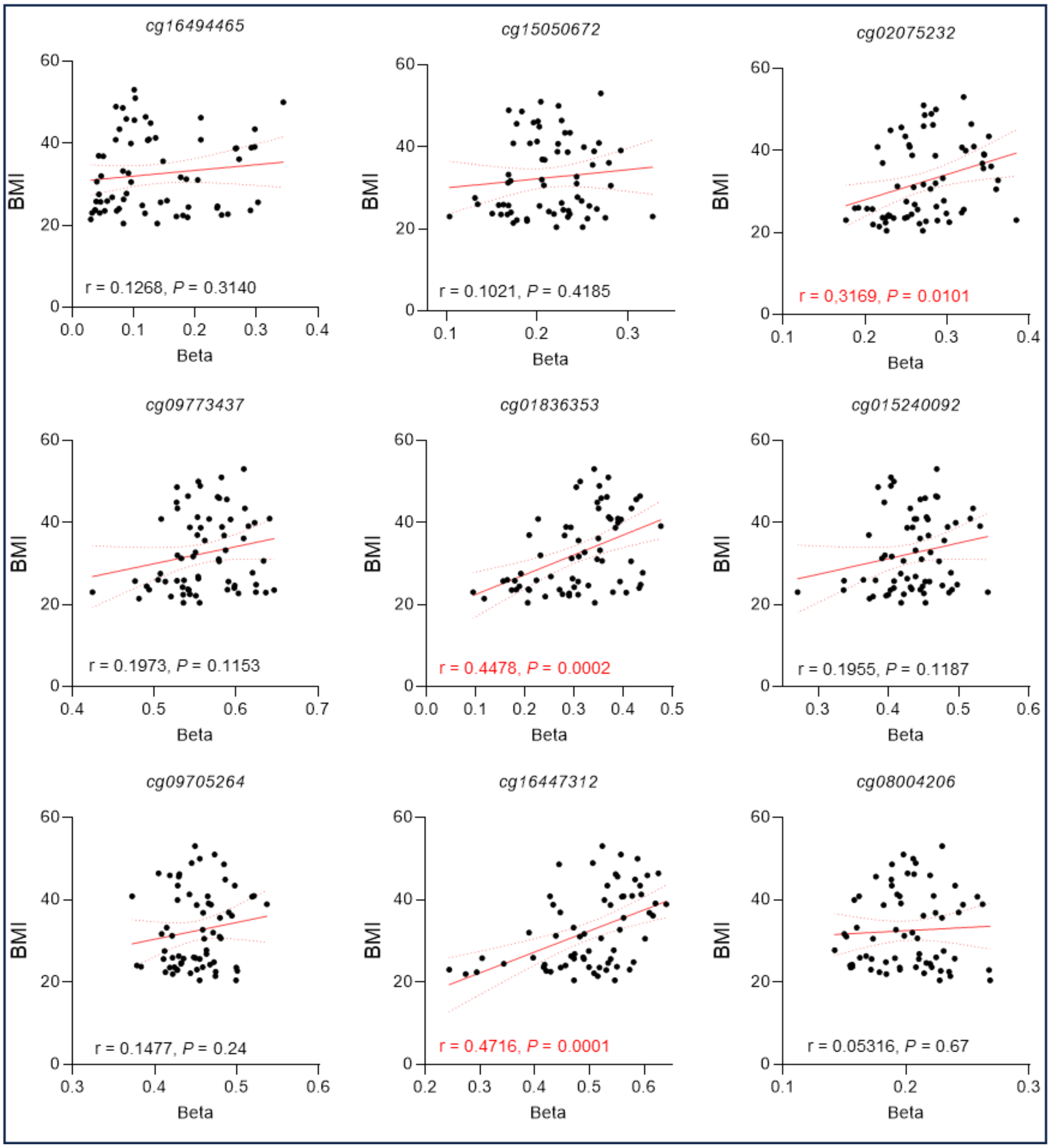
cg16494465
r = 0.1268, P = 0.3140
cg15050672
r = 0.1021, P = 0.4185
cg02075232
r = 0,3169, P = 0.0101
cg09773437
r = 0.1973, P = 0.1153
cg01836353
r = 0.4478, P = 0.0002
cg015240092
r = 0.1955, P = 0.1187
cg09705264
r = 0.1477, P = 0.24
cg16447312
r = 0.4716, P = 0.0001
cg08004206
r = 0.05316, P = 0.67
BMI
Beta

Supplementary Figure 3: Impact of AQP7 deficiency and glucose consumption on inflammation.

(A,D) AQP7-deficient mice exhibit significantly elevated MCP-1 levels both locally and systemically. **(B,E)** IL-6 levels are also markedly increased in these mice at both local and systemic levels. **(C, F)** High glucose diet further enhances systemic IL-1β levels across all mice compared to controls**.**

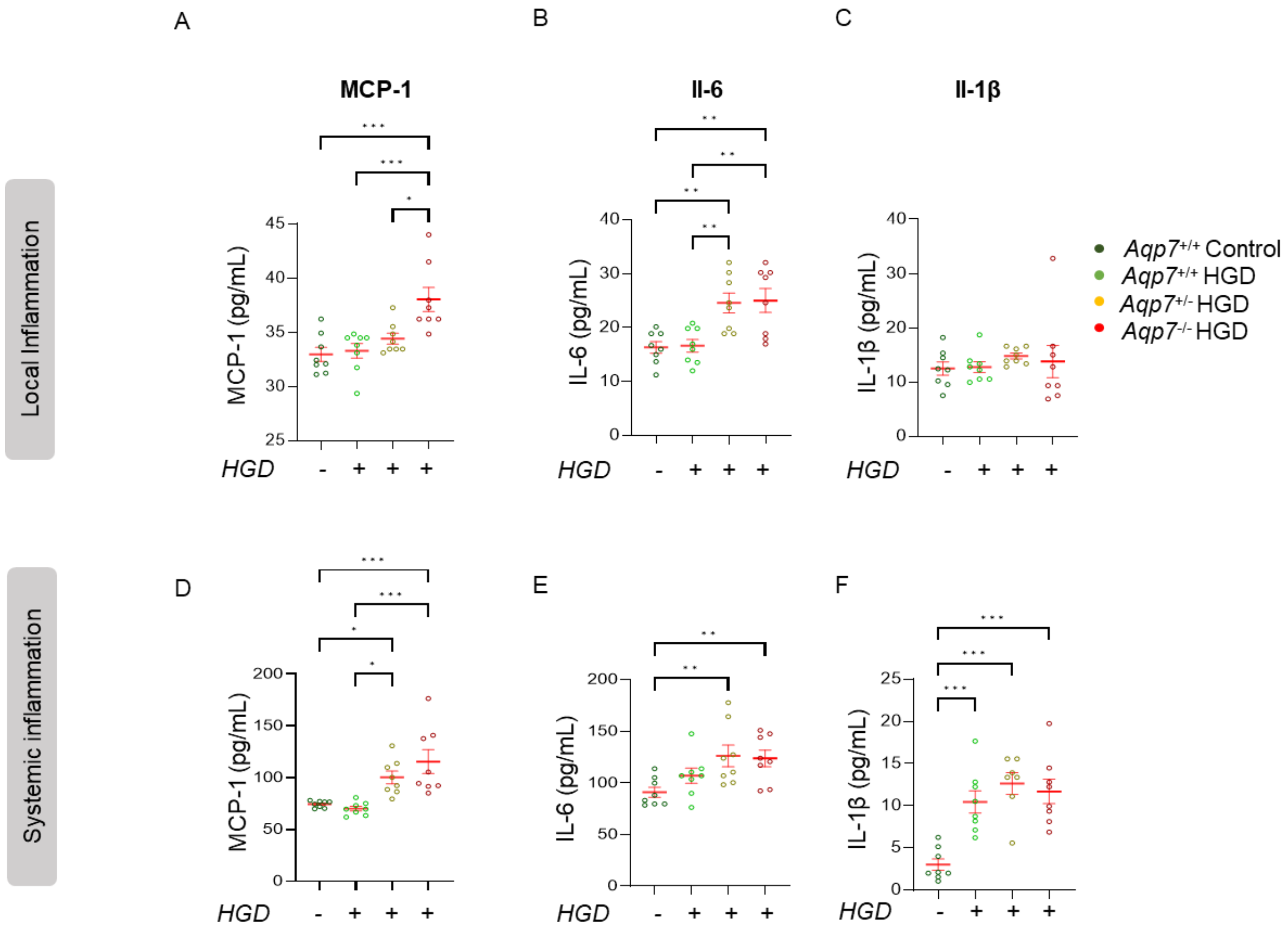

Supplementary Figure 4: Timeline of adipogenesis.

Schematic representation of the adipogenesis timeline in MEF-derived adipocytes, detailing key phases from induction to full maturation. The process begins with the harvesting and culture of mouse embryonic fibroblasts. At confluence (Day 0), cells are induced to differentiate with an adipogenic cocktail, which activates key transcription factors and initiates the process of mitotic clonal expansion. During the early phase (Day 1-2), cells undergo an initial commitment to adipogenesis, marked by changes in cellular morphology and the early expression of adipogenic transcription factors. By day 3–6, lipid droplets start to form, and key adipogenic markers are increasingly expressed. As differentiation progresses (Day 7–9), the lipid droplets grow larger, and adipokine secretion begins. Fully mature adipocytes (Day 10–12) exhibit large lipid droplets, enhanced metabolic activity, and peak expression of adipokines and lipolytic enzymes.

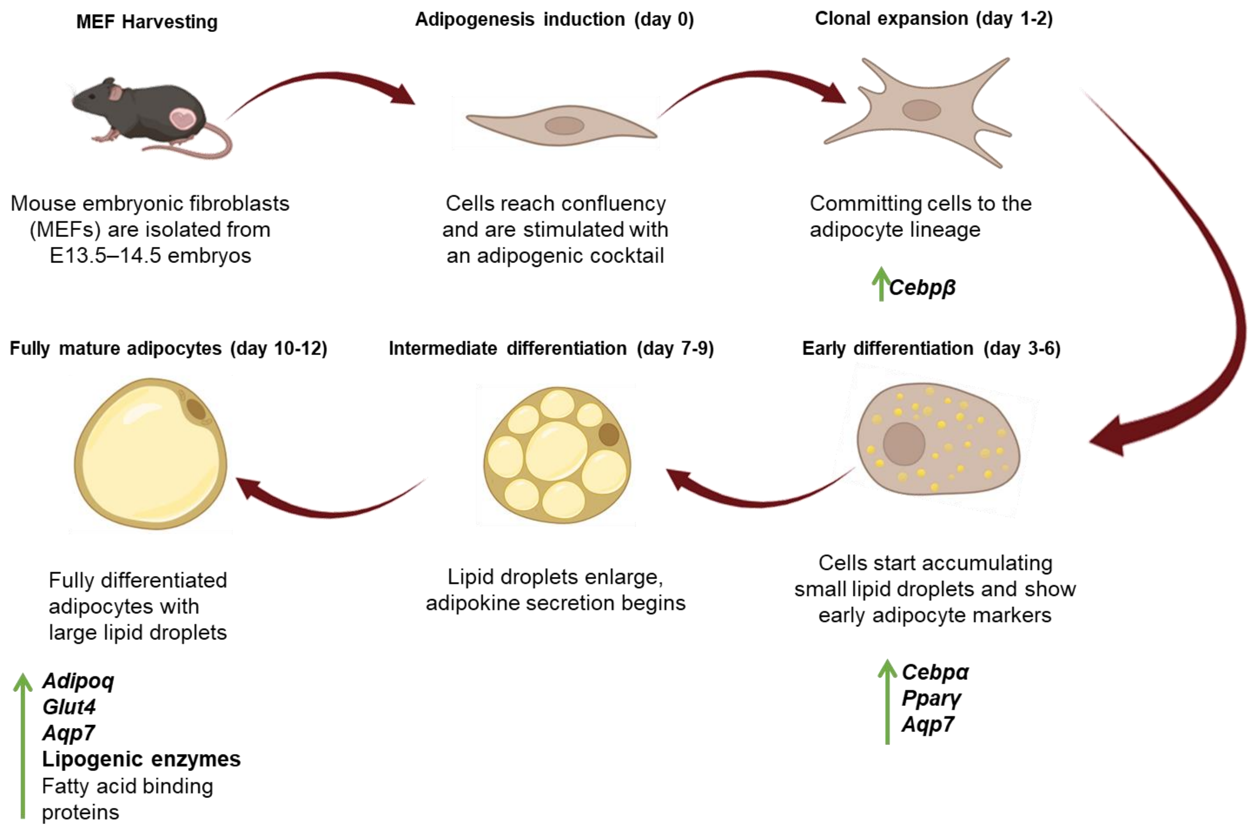

Supplementary Figure 5: Workflow of the secretome analysis.

A schematic representation of the secretome analysis process. Supernatants are collected from adipocyte cultures at specific time points, representing the proteins released into the extracellular environment. These proteins are then profiled using liquid chromatography mass spectrometry (LC/MS) to identify and quantify the secreted factors. The detected proteins are analyzed and compared against a secreted protein database (Mouse Uniprot annotated as secreted) to filter out non-secreted proteins and ensure that only secreted factors are considered. To ensure accurate comparison, proteins showing significant differences prior to adipocyte induction are excluded from the analysis. The remaining proteins are then analysed for changes in secretion profiles, followed by pathway analysis to identify enriched biological processes and signalling pathways.

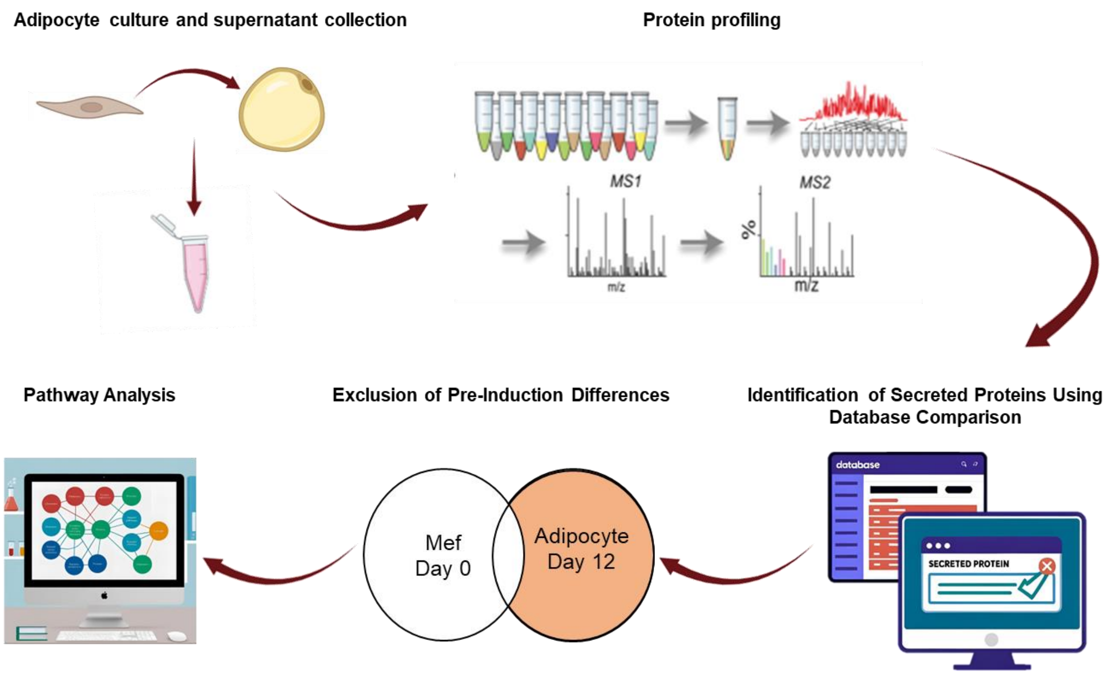

Supplementary Figure 6: Secretome profiling of adipocyte maturation.

(A) Detailed analysis revealed 30 upregulated and 129 downregulated proteins in the *Aqp7*$^{-/-}$ secretory profile. For this analysis, proteins that were significantly regulated ($p<0.05$) at day 0; in the same direction as on d12 were considered "KO background changes" and removed for the analysis. Remaining proteins indicate specific alterations in the secretion patterns during adipocyte maturation in the absence of AQP7 In red top 30 most upregulated proteins, in blue top 30 most downregulated proteins in the secretome. **(B)** Pathway enrichment analysis of the potential interactions of these secreted proteins with diverse cell types within the VAT (endothelial cells, macrophages, fibroblasts, and adipocytes) demonstrated significant differences in the associated biological processes, highlighting the functional consequences of *Aqp7* loss on adipocyte-secreted signals and their impact on the surrounding microenvironment. **(C)** Validation of the main altered pathways at the transcript level in *Aqp7*-deficient VAT.

A

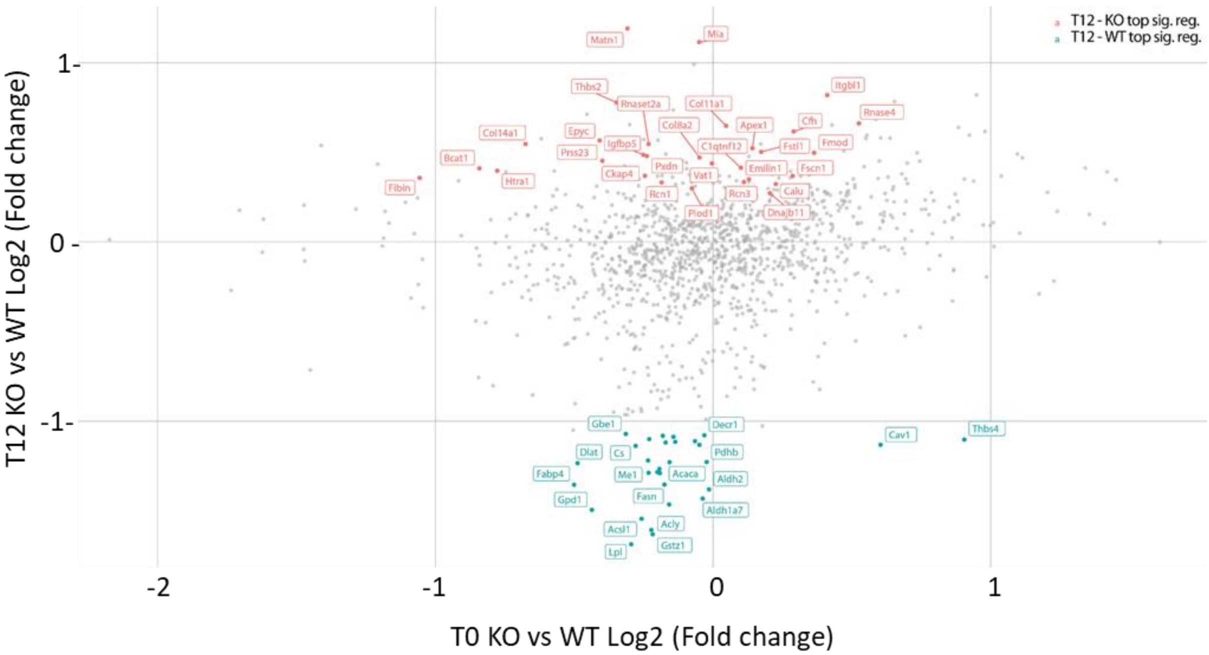

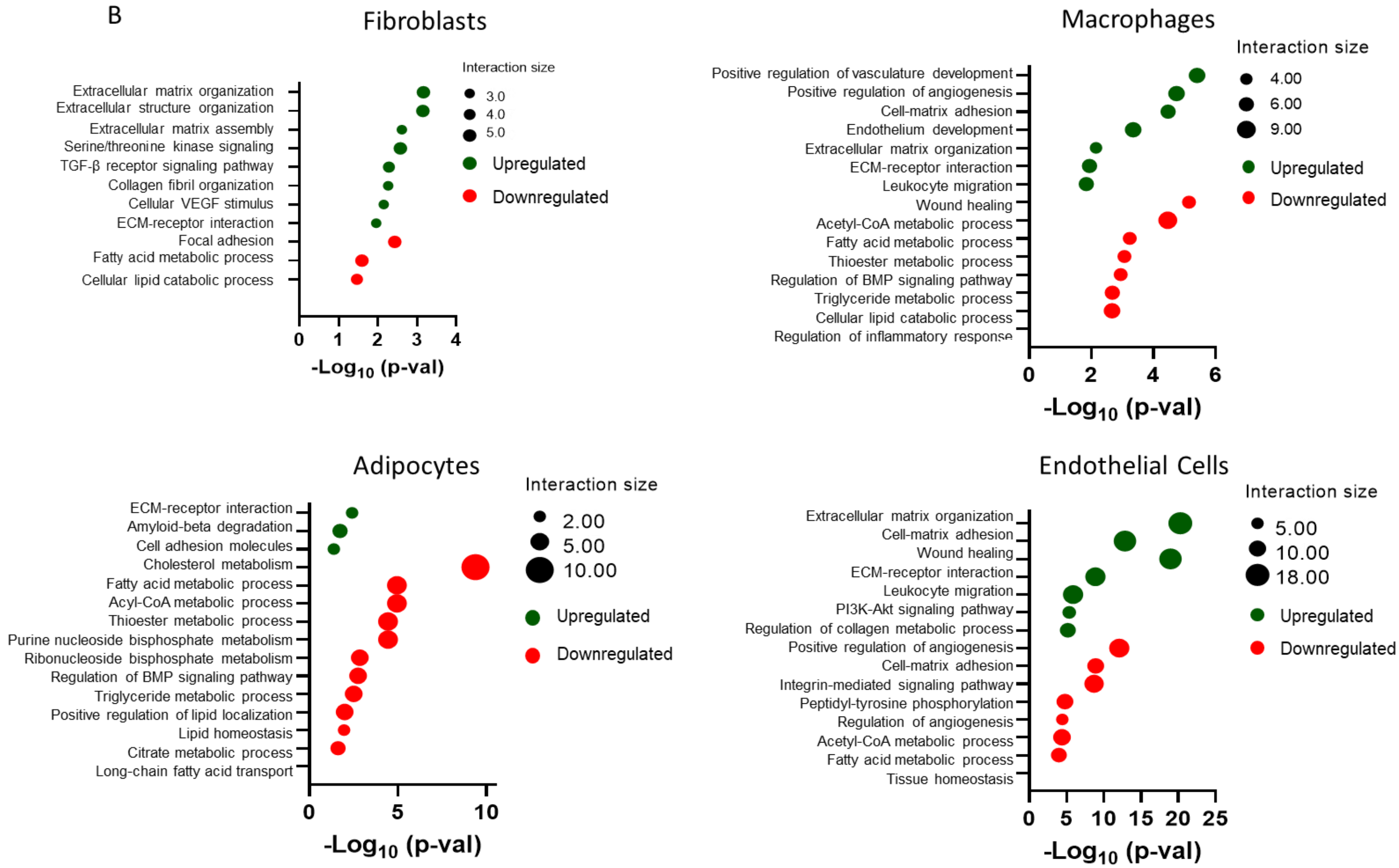
B
Fibroblasts
Interaction size
3.0
4.0
5.0
Upregulated
Downregulated
Extracellular matrix organization
Extracellular structure organization
Extracellular matrix assembly
Serine/threonine kinase signaling
TGF-β receptor signaling pathway
Collagen fibril organization
Cellular VEGF stimulus
ECM-receptor interaction
Focal adhesion
Fatty acid metabolic process
Cellular lipid catabolic process
0 1 2 3 4
-Log10 (p-val)
Macrophages
Interaction size
4.00
6.00
9.00
Upregulated
Downregulated
Positive regulation of vasculature development
Positive regulation of angiogenesis
Cell-matrix adhesion
Endothelium development
Extracellular matrix organization
ECM-receptor interaction
Leukocyte migration
Wound healing
Acetyl-CoA metabolic process
Fatty acid metabolic process
Thioester metabolic process
Regulation of BMP signaling pathway
Triglyceride metabolic process
Cellular lipid catabolic process
Regulation of inflammatory response
0 2 4 6
-Log10 (p-val)
Adipocytes
Interaction size
2.00
5.00
10.00
Upregulated
Downregulated
ECM-receptor interaction
Amyloid-beta degradation
Cell adhesion molecules
Cholesterol metabolism
Fatty acid metabolic process
Acyl-CoA metabolic process
Thioester metabolic process
Purine nucleoside bisphosphate metabolism
Ribonucleoside bisphosphate metabolism
Regulation of BMP signaling pathway
Triglyceride metabolic process
Positive regulation of lipid localization
Lipid homeostasis
Citrate metabolic process
Long-chain fatty acid transport
0 5 10
-Log10 (p-val)
Endothelial Cells
Interaction size
5.00
10.00
18.00
Upregulated
Downregulated
Extracellular matrix organization
Cell-matrix adhesion
Wound healing
ECM-receptor interaction
Leukocyte migration
PI3K-Akt signaling pathway
Regulation of collagen metabolic process
Positive regulation of angiogenesis
Cell-matrix adhesion
Integrin-mediated signaling pathway
Peptidyl-tyrosine phosphorylation
Regulation of angiogenesis
Acetyl-CoA metabolic process
Fatty acid metabolic process
Tissue homeostasis
0 5 10 15 20 25
-Log10 (p-val)

C

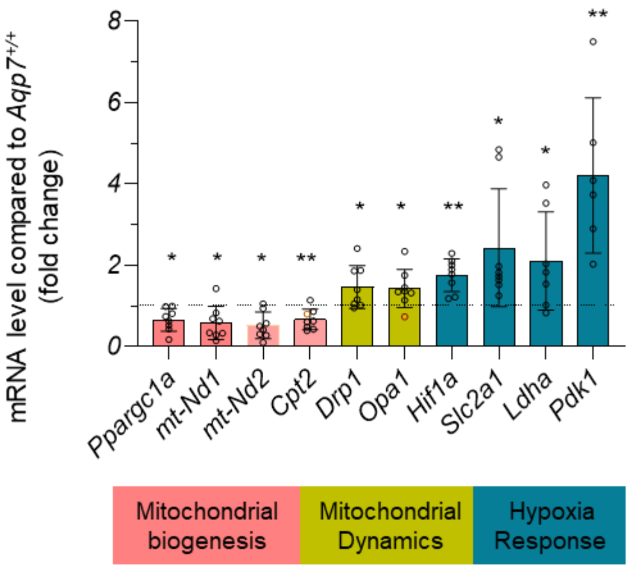
mRNA level compared to Aqp7+/+
(fold change)
8
6
4
2
0
**
*
*
*
*
**
*
*
**
*
*
Ppargc1a
mt-Nd1
mt-Nd2
Cpt2
Drp1
Opa1
Hif1a
Slc2a1
Ldha
Pdk1
Mitochondrial biogenesis
Mitochondrial Dynamics
Hypoxia Response

Supplementary Figure 7: Uncropped Western blot membranes.

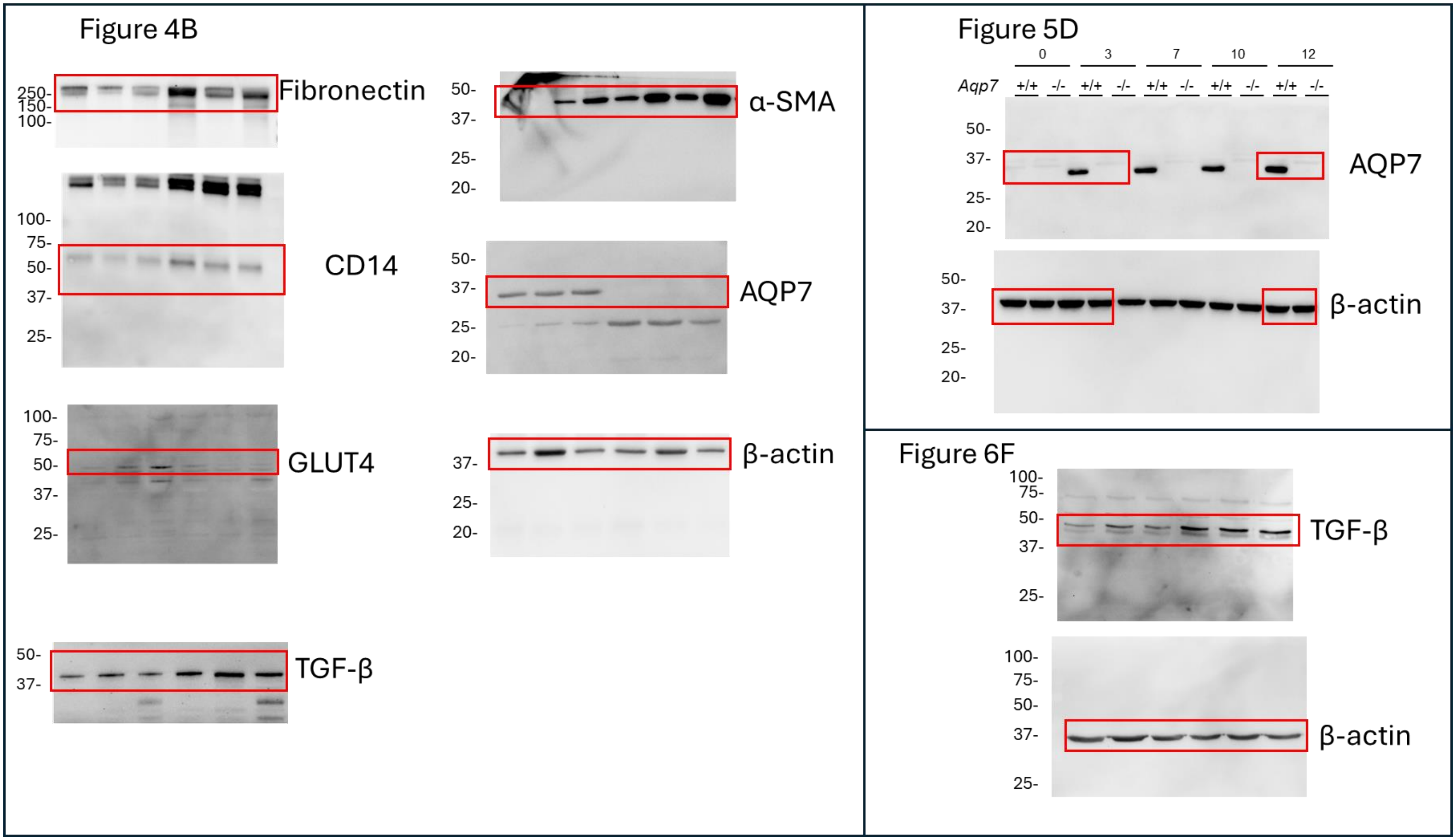

Supplement: Supplementary file 1 — Supplementary Information [file 44324_2025_85_MOESM1_ESM.pdf]
